# Is the frequency of breakfast consumption associated with life satisfaction in children and adolescents? A cross-sectional study with 154,151 participants from 42 countries

**DOI:** 10.1186/s12937-024-00979-5

**Published:** 2024-07-16

**Authors:** José Francisco López-Gil, Mark A. Tully, Carlos Cristi-Montero, Javier Brazo-Sayavera, Anelise Reis Gaya, Joaquín Calatayud, Rubén López-Bueno, Lee Smith

**Affiliations:** 1 One Health Research Group, Universidad de Las Americas, Quito, Ecuador; 2 School of Medicine, Ulster University, Londonderry, Northern Ireland UK; 3 Physical Education School, IRyS Group, Pontificia Universidad Católica de Valparaíso, Valparaíso, 2530388 Chile; 4 Department of Sports and Computer Science, Universidad Pablo de Olavide, Seville, Spain; 5 Federal University of Rio Grande do Sul (UFRGS), Rua Felizardo, n° 750 - Jardim Botânico, Porto Alegre, RS Brazil; 6 National Research Centre for the Working Environment, Copenhagen, Denmark; 7 Department of Physiotherapy, Exercise Intervention for Health Research Group (EXINH-RG), University of Valencia, Valencia, Spain; 8 Department of Physical Medicine and Nursing, University of Zaragoza, Zaragoza, Spain; 9 Centre for Health Performance and Wellbeing, Anglia Ruskin University, Cambridge, UK

**Keywords:** Eating healthy, Well-being, Mental health, Youth, Adolescence, Childhood

## Abstract

**Background:**

The promotion of daily breakfast consumption and the importance of making appropriate breakfast choices have been underscored as significant public health messages. The aim of this study was to examine the relationship between breakfast frequency and life satisfaction in large and representative samples of school-going children and adolescents aged 10–17 years from 42 different countries.

**Methods:**

This study used information from the 2017/2018 Health Behavior in School-aged Children study, comprising nationally representative samples of children and adolescents aged 10–17 years who were attending school. The total number of participants from the 42 countries included in the study was 155,451 (51.3% girls). The evaluation of breakfast consumption in this study involved a specific question: “How often do you typically have breakfast (more than a glass of milk or fruit juice)?”. To measure life satisfaction, a subjective assessment scale was used in the form of a ladder, visually spanning from 0 to 10. On this scale, the topmost point (10) denotes the highest conceivable quality of life, whereas the bottom point (0) represents the worst imaginable quality of life.

**Results:**

After adjusting for several covariates, the lowest estimated marginal mean of life satisfaction was identified in those participants who skipped breakfast (mean [M] = 5.6, 95% confidence interval [CI] 5.5 to 5.8). Conversely, the highest estimated marginal mean of life satisfaction was observed in those who had breakfast every day (M = 6.5, 95% CI 6.3 to 6.6). Overall, a nearly linear relationship between higher frequency of breakfast and greater life satisfaction in children and adolescents was identified (*p*-for-trend < 0.001). In addition, the highest estimated marginal mean of life satisfaction score was identified in those participants from Portugal who had breakfast every day (M = 7.7; 95% CI 6.9 to 8.5 points). Conversely, the lowest estimated marginal mean of life satisfaction was observed in those participants from Romania who no breakfast (M = 3.5; 95% CI 2.6 to 4.4 points).

**Conclusions:**

There is a nearly linear relationship between higher frequency of breakfast and greater life satisfaction in children and adolescents. Considering the potential health advantages associated with breakfast during this critical age phase, these findings imply the necessity for additional global efforts to promote increased breakfast consumption among children and adolescents.

**Supplementary Information:**

The online version contains supplementary material available at 10.1186/s12937-024-00979-5.

## Introduction

Life satisfaction is commonly defined as an individual’s cognitive and emotional assessment of their own life [1]. While extensive research has been conducted on this topic among adults, comparatively less focus has been placed on children and adolescents [2, 3]. However, there is a growing interest in understanding subjective well-being, including life satisfaction, among children and adolescents because of the significant changes that occur during this critical period [4, 5]. Some studies have suggested a notable decline in well-being during adolescence [4, 6–8], which is attributed to associated biological and psychosocial changes [9]. Therefore, at the public health level, it is essential to identify the factors that positively influence life satisfaction, a crucial cognitive aspect of hedonic subjective well-being [10]. Adolescent well-being is significant for both personal and societal reasons, given that adolescence is a pivotal life stage in which crucial elements contributing to lifelong well-being are either established or neglected [11]. Consequently, recognizing these factors is particularly important given the potential impact of adolescent well-being on later adult mental health [12].

While there is a general consensus in academic circles that child and adolescent well-being is a complex concept with multiple dimensions [13], scientific literature emphasizes the significance of lifestyle as a crucial factor [14–20]. Numerous studies have established a connection between overall diet and well-being [15, 16], with diet quality being linked to life satisfaction among young individuals [19]. For instance, a study involving Chilean children found that higher adherence to the Mediterranean diet was associated with increased life satisfaction [19]. Another relevant aspect of adolescence is the habit of consuming breakfast [16, 21–24]. Regularly having a nutritious breakfast has been associated with numerous positive effects on psychosocial and health-related behaviors, including enhanced memory recall, improved cognitive function, and increased levels of physical activity, among other benefits [21, 24, 25]. Conversely, a meta-analysis found that skipping breakfast was positively associated with the odds of depression, stress, and psychological distress in all age groups and anxiety in adolescence, underlining the role of breakfast on mental health [16]. Despite this, the association of breakfast frequency with adolescents’ perceived life satisfaction has been relatively underexplored [26, 27]. In the context of adolescence, breakfast quality plays a crucial role in the interplay between lifestyle and psychosocial health during early adolescence [24, 28]. Recommendations suggest that a healthy breakfast should encompass a variety of foods from different food groups, such as fruits, vegetables, whole grains, lean protein, and healthy fats, while avoiding sugary cereals and processed foods [29]. There is also a preference for the consumption of whole fruits over fruit juices due to more conclusive evidence regarding the health benefits of whole fruits [30]. Consequently, the promotion of daily breakfast consumption and the importance of making appropriate breakfast choices have been underscored as significant public health messages [31]. Despite this, it is noteworthy that many adolescents tend to skip breakfast, and the prevalence of children and adolescents skipping this important meal is increasing [32–34]. Given these considerations, the aim of this study was to examine the relationship between breakfast frequency and life satisfaction in large and representative samples of school-going children and adolescents aged 10–17 years from 42 different countries.

## Methods

### Study design and population

This cross-sectional study included data from 42 countries, including Albania, Armenia, Austria, Azerbaijan, Belgium (Flanders and Wallonia), Bulgaria, Canada, Croatia, the Czech Republic, Denmark, England, Estonia, Finland, France, Georgia, Germany, Greece, Greenland, Hungary, Iceland, Israel, Italy, Kazakhstan, Latvia, Lithuania, Luxembourg, Malta, the Netherlands, North Macedonia, Norway, Poland, Portugal, the Republic of Moldova, Romania, the Russian Federation, Scotland, Serbia, Slovenia, Spain, Sweden, Ukraine, and Wales. This study used information from the 2017/2018 Health Behavior in School-aged Children (HBSC) study [35], comprising nationally representative samples of children and adolescents aged 10–17 years who were attending school. Slovakia was removed because of missing information on frequency of breakfast consumption (independent variable). Ireland and Switzerland were excluded from the analysis due to insufficient information on frequency of family meals (covariate). The total number of participants from the 42 countries included in the study was 155,451 (51.3% girls). The selection of children and adolescents for the study involved a random sampling method from various schools. The participants completed a standardized test anonymously (i.e., self-reported), and the assessment was conducted in their native language. Students had the option to decline answering specific questions. Each participating country obtained institutional ethics approval and written informed consent forms were signed by the schools, children, adolescents, and their parents or legal guardians. Notably, because the present study entailed a secondary analysis of anonymized data, formal approval from an ethics committee was deemed unnecessary.

### Procedures

#### Frequency of breakfast consumption (independent variable)

The evaluation of breakfast consumption in this study involved a specific question: “How often do you typically have breakfast (more than a glass of milk or fruit juice)?” [35]. Participants were asked to respond to this question separately for weekdays, with response options ranging from 0 to 5 days, and weekends, with options ranging from 0 to 2 days. The total frequency of breakfast consumption by the children and adolescents was calculated by summing the number of days they reported having breakfast.

#### Life satisfaction (dependent variable)

To measure life satisfaction, a subjective assessment scale was used in the form of a ladder, visually spanning from 0 to 10 [36]. On this scale, the topmost point (10) denotes the highest conceivable quality of life, whereas the bottom point (0) represents the worst imaginable quality of life. Participants were directed to place an “X” adjacent to the number that most accurately reflected their present position on the life satisfaction scale. This visual method offered a graphic representation of the participants’ self-assessments of their current level of life satisfaction.

#### Covariates

The children and adolescents in this study provided self-reported information regarding their age and sex. The Family Affluence Scale (FAS-III) was employed to assess the socioeconomic status (SES), consisting of six questions with responses ranging from 0 to 13 points [37]. Total scores obtained by summing individual responses reflected a higher SES with a higher score. The FAS-III covered various aspects of family material assets, including the number of bathrooms, cars, non-shared bedrooms, dishwashers, computers, and foreign vacations taken in the last 12 months. *Ridit* scores, specific to age groups and sex, were calculated for each participating country according to international standards [38]. These *ridit* scores categorized children and adolescents into three SES groups: bottom 20% (low SES), middle 60% (medium SES), and top 20% (high SES). The frequency of different eating habits was determined through questions such as “How many times a week do you eat fruits/vegetables/sweets/soft drinks?” with response options ranging from never to more than once per day. The frequency of family meals was assessed using the question: “How often do you and your family typically have meals together?” with response options including “every day”, “most days”, “approximately once a week”, “less often”, and “never”. Physical activity was evaluated with the question “In a typical or usual week, how many days do you engage in physical activity for at least 60 minutes a day?” with responses ranging from 0 to 7 days per week. Finally, children and adolescents self-reported their body weight and height, which were then used to calculate their body mass index (kg/m^2^). The selection of the covariates included was based in the scientific literature [26, 39, 40].

### Statistical analysis

The data in this study are presented as counts and percentages for categorical variables and as means and standard deviations for continuous variables. Generalized linear mixed models were employed to investigate the relationship between the frequency of breakfast consumption (as a fixed effect), country (as a random effect), and life satisfaction (as the outcome). The data were weighted by expansion factors to ensure the representativeness of the country. Furthermore, these associations were further adjusted for various covariates, including sex, age group, SES, fruit consumption, vegetable consumption, soft drink consumption, sweet consumption, frequency of family meals, physical activity, and body mass index. To determine, how much variability there is between individuals across all countries, the intraclass correlation coefficient was computed. Additionally, estimated marginal means of life satisfaction and their 95% confidence intervals (CIs) were calculated based on the frequency of breakfast consumption. As a sensitivity analysis and to observe the consistency of the results regardless of the country examined, the estimated marginal means of life satisfaction (adjusted for the same covariates) of the two extremes of breakfast frequency (i.e., 0 days versus 7 days) were shown. Furthermore, to address potential bias stemming from incomplete data, a sensitivity analysis using multiple imputation techniques was applied, assuming that missing data occurred randomly. The *mice* package was used to replace missing values through chained equations [41]. To ensure robustness, 36 datasets were created with multiple imputations, in line with recommendations to set the number of imputations to be more than 100 times the highest proportion of missing information (i.e., 36.0%) [42].

## Results

Table 1 shows the descriptive data of the participants according to the frequency of breakfast consumption by the listwise deletion method. Overall, the life satisfaction mean was 7.8 ± 1.9 points. Regarding the frequency of breakfast consumption, participants who skipped breakfast (i.e., 0 times per week) showed the lowest mean life satisfaction (M = 6.9 ± 2.4 points), while the highest mean was observed in those who had breakfast daily (i.e., seven times per week) (M = 8.1 ± 1.7 points).

**Table 1:** Descriptive data of the study participants according to frequency of breakfast consumption by listwise deletion method. (*N* = 154,151)

Variables		Frequency of breakfast consumption (days)								
		0	1	2	3	4	5	6	7	Total
Age	Mean (SD)	14.2 (1.6)	14.1 (1.6)	13.8 (1.6)	13.7 (1.6)	13.7 (1.6)	13.7 (1.6)	13.6 (1.6)	13.4 (1.7)	13.6 (1.7)
Age group	Aged 10–12 years	879 (15.9)	1197 (16.8)	3858 (21.7)	1846 (24.0)	2102 (23.8)	3032 (23.7)	3655 (26.0)	25,154 (31.1)	41,723 (27.0)
	Aged 13–15 years	2031 (36.8)	2850 (40.1)	7176 (40.3)	3070 (40.0)	3734 (42.3)	5153 (40.2)	5737 (40.8)	32,519 (40.2)	62,271 (40.3)
	Aged 16–17 years	2605 (47.2)	3066 (43.1)	6761 (38.0)	2766 (36.0)	2993 (33.9)	4622 (36.1)	4685 (33.3)	23,184 (28.7)	50,681 (32.8)
Sex	Boys (%)	2599 (47.1)	2935 (41.3)	7558 (42.5)	3487 (45.4)	4082 (46.2)	6237 (48.7)	7130 (50.7)	41,297 (51.1)	75,327 (48.7)
	Girls (%)	2916 (52.9)	4178 (58.7)	10,237 (57.5)	4195 (54.6)	4746 (53.8)	6570 (51.3)	6947 (49.3)	39,560 (48.9)	79,349 (51.3)
SES	Low SES (%)	1400 (25.4)	1626 (22.9)	3765 (21.2)	1650 (21.5)	1810 (20.5)	2485 (19.4)	2550 (18.1)	12,962 (16.0)	28,249 (18.3)
	Medium SES (%)	3260 (59.1)	4338 (61.0)	10,991 (61.8)	4772 (62.1)	5401 (61.2)	8042 (62.8)	8706 (61.8)	50,584 (62.6)	96,093 (62.1)
	High SES (%)	855 (15.5)	1149 (16.2)	3039 (17.1)	1260 (16.4)	1618 (18.3)	2280 (17.8)	2822 (20.0)	17,311 (21.4)	30,334 (19.6)
Fruits consumption	Never (%)	373 (6.8)	291 (4.1)	525 (3.0)	281 (3.7)	213 (2.4)	360 (2.8)	325 (2.3)	1680 (2.1)	4049 (2.6)
	Less once a week (%)	648 (11.7)	805 (11.3)	1271 (7.1)	530 (6.9)	536 (6.1)	813 (6.4)	786 (5.6)	3736 (4.6)	9126 (5.9)
	Once a week (%)	695 (12.6)	925 (13.0)	1989 (11.2)	880 (11.5)	939 (10.6)	1156 (9.0)	1316 (9.4)	6190 (7.7)	14,090 (9.1)
	2–4 days a week (%)	1348 (24.4)	1994 (28.0)	4862 (27.3)	2153 (28.0)	2662 (30.2)	3691 (28.8)	3898 (27.7)	19,112 (23.6)	39,721 (25.7)
	5–6 days a week (%)	708 (12.8)	954 (13.4)	2664 (15.0)	1138 (14.8)	1473 (16.7)	2144 (16.7)	2497 (17.7)	13,213 (16.3)	24,790 (16.0)
	Once daily (%)	704 (12.8)	991 (13.9)	2903 (16.3)	1192 (15.5)	1350 (15.3)	2123 (16.6)	2454 (17.4)	15,787 (19.5)	27,503 (17.8)
	More than once daily (%)	1040 (18.9)	1152 (16.2)	3580 (20.1)	1509 (19.6)	1654 (18.7)	2521 (19.7)	2801 (19.9)	21,140 (26.1)	35,398 (22.9)
Vegetables consumption	Never (%)	523 (9.5)	480 (6.7)	890 (5.0)	385 (5.0)	379 (4.3)	514 (4.0)	497 (3.5)	2514 (3.1)	6182 (4.0)
	Less once a week (%)	625 (11.3)	652 (9.2)	1319 (7.4)	615 (8.0)	615 (7.0)	780 (6.1)	771 (5.5)	3607 (4.5)	8983 (5.8)
	Once a week (%)	667 (12.1)	904 (12.7)	2018 (11.3)	926 (12.1)	1053 (11.9)	1323 (10.3)	1323 (9.4)	6148 (7.6)	14,361 (9.3)
	2–4 days a week (%)	1222 (22.2)	1830 (25.7)	4326 (24.3)	1899 (24.7)	2355 (26.7)	3281 (25.6)	3465 (24.6)	17,389 (21.5)	35,766 (23.1)
	5–6 days a week (%)	814 (14.8)	1187 (16.7)	3235 (18.2)	1292 (16.8)	1661 (18.8)	2537 (19.8)	2949 (20.9)	15,654 (19.4)	29,328 (19.0)
	Once daily (%)	768 (13.9)	1093 (15.4)	3226 (18.1)	1323 (17.2)	1477 (16.7)	2373 (18.5)	2789 (19.8)	17,440 (21.6)	30,488 (19.7)
	More than once daily (%)	896 (16.3)	969 (13.6)	2781 (15.6)	1243 (16.2)	1290 (14.6)	1999 (15.6)	2284 (16.2)	18,106 (22.4)	29,567 (19.1)
Soft drinks consumption	Never (%)	813 (14.7)	864 (12.1)	2204 (12.4)	1075 (14.0)	1096 (12.4)	1549 (12.1)	1913 (13.6)	12,733 (15.7)	22,246 (14.4)
	Less once a week (%)	1078 (19.6)	1441 (20.3)	3859 (21.7)	1754 (22.8)	1998 (22.6)	3038 (23.7)	3456 (24.5)	21,625 (26.7)	38,250 (24.7)
	Once a week (%)	783 (14.2)	1266 (17.8)	3145 (17.7)	1450 (18.9)	1722 (19.5)	2494 (19.5)	2790 (19.8)	16,180 (20.0)	29,831 (19.3)
	2–4 days a week (%)	937 (17.0)	1451 (20.4)	3597 (20.2)	1475 (19.2)	1831 (20.7)	2658 (20.8)	2834 (20.1)	14,872 (18.4)	29,656 (19.2)
	5–6 days a week (%)	513 (9.3)	669 (9.4)	1533 (8.6)	649 (8.4)	792 (9.0)	1141 (8.9)	1216 (8.6)	5452 (6.7)	11,966 (7.7)
	Once daily (%)	496 (9.0)	558 (7.8)	1475 (8.3)	573 (7.5)	625 (7.1)	878 (6.9)	888 (6.3)	4415 (5.5)	9908 (6.4)
	More than once daily (%)	895 (16.2)	865 (12.2)	1982 (11.1)	706 (9.2)	765 (8.7)	1047 (8.2)	981 (7.0)	5579 (6.9)	12,819 (8.3)
Sweets consumption	Never (%)	384 (7.0)	313 (4.4)	697 (3.9)	370 (4.8)	354 (4.0)	446 (3.5)	512 (3.6)	3059 (3.8)	6134 (4.0)
	Less once a week (%)	844 (15.3)	979 (13.8)	2201 (12.4)	990 (12.9)	1128 (12.8)	1527 (11.9)	1682 (11.9)	10,197 (12.6)	19,547 (12.6)
	Once a week (%)	875 (15.9)	1232 (17.3)	2981 (16.8)	1395 (18.2)	1571 (17.8)	2362 (18.4)	2762 (19.6)	15,135 (18.7)	28,314 (18.3)
	2–4 days a week (%)	1378 (25.0)	1826 (25.7)	4698 (26.4)	1968 (25.6)	2582 (29.2)	3822 (29.8)	4139 (29.4)	22,471 (27.8)	42,884 (27.7)
	5–6 days a week (%)	569 (10.3)	928 (13.0)	2324 (13.1)	1111 (14.5)	1243 (14.1)	1636 (12.8)	1866 (13.3)	10,207 (12.6)	19,884 (12.9)
	Once daily (%)	592 (10.7)	845 (11.9)	2426 (13.6)	937 (12.2)	990 (11.2)	1541 (12.0)	1687 (12.0)	10,120 (12.5)	19,139 (12.4)
	More than once daily (%)	872 (15.8)	990 (13.9)	2469 (13.9)	910 (11.8)	961 (10.9)	1473 (11.5)	1431 (10.2)	9668 (12.0)	18,774 (12.1)
Family meals	Never (%)	489 (8.9)	331 (4.6)	549 (3.1)	205 (2.7)	215 (2.4)	307 (2.4)	243 (1.7)	941 (1.2)	3280 (2.1)
	Less often (%)	713 (12.9)	838 (11.8)	1554 (8.7)	616 (8.0)	692 (7.8)	887 (6.9)	834 (5.9)	3470 (4.3)	9603 (6.2)
	About once a week (%)	590 (10.7)	986 (13.9)	2151 (12.1)	853 (11.1)	1029 (11.7)	1350 (10.5)	1275 (9.1)	5484 (6.8)	13,718 (8.9)
	Most days (%)	1406 (25.5)	2249 (31.6)	5665 (31.8)	2514 (32.7)	3145 (35.6)	4541 (35.5)	5213 (37.0)	25,628 (31.7)	50,361 (32.6)
	Every day (%)	2318 (42.0)	2710 (38.1)	7877 (44.3)	3494 (45.5)	3747 (42.4)	5722 (44.7)	6513 (46.3)	45,333 (56.1)	77,714 (50.2)
Weekly physical activity (days) ^†^	Mean (SD)	3.6 (2.3)	3.6 (2.1)	3.8 (2.1)	3.8 (2.1)	3.9 (2.0)	4.0 (2.0)	4.1 (2.0)	4.3 (2.0)	5.1 (2.1)
Body mass index (kg/m^2^)	Mean (SD)	20.5 (4.0)	20.6 (3.9)	20.2 (3.7)	20.0 (3.8)	19.9 (3.7)	19.9 (3.7)	19.6 (3.5)	19.1 (3.4)	19.6 (3.6)
Life satisfaction (score)	Mean (SD)	6.9 (2.4)	7.0 (2.2)	7.5 (2.0)	7.5 (2.0)	7.5 (1.9)	7.6 (1.9)	7.7 (1.8)	8.1 (1.7)	7.8 (1.9)

*SD* standard deviation, *SES* socioeconomic status. ^†^At least 60 min a day

**Fig. 1:**
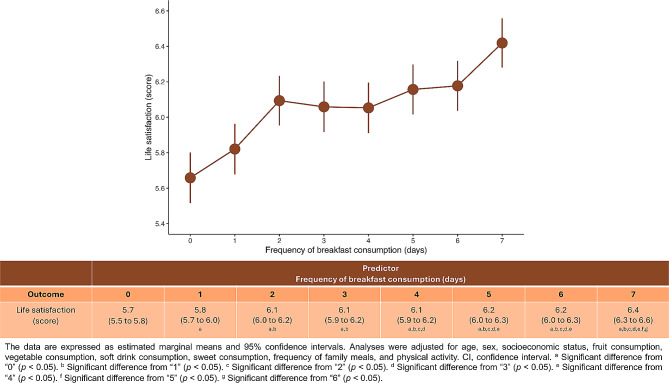
Estimated marginal means of life satisfaction based on the frequency of breakfast consumption by listwise deletion method. Data expressed as dots (means) and lines (95% confidence intervals). Adjusted for sex, age group, socioeconomic status, fruit consumption, vegetable consumption, soft drink consumption, sweet consumption, breakfast consumption, frequency of family meals, physical activity, and body mass index

Figure 1 shows the estimated marginal means of life satisfaction according to the frequency of breakfast consumption by the listwise deletion method. The lowest estimated marginal mean of life satisfaction was identified in those participants who no breakfast (mean [M] = 5.7, 95% CI 5.5 to 5.8 points). Conversely, the highest estimated marginal mean of life satisfaction was observed in those who had breakfast every day (M = 6.4, 95% CI 6.3 to 6.6 points). Overall, a nearly linear relationship between higher frequency of breakfast and greater life satisfaction in children and adolescents was identified (*p*-for-trend < 0.001). The analysis reporting the estimated marginal means of life satisfaction according to the frequency of breakfast consumption using multiple imputations by chained methods can be found in Figure S1. Moreover, Table S1 and Table S2 display the full results of the generalized linear models assessing the association between frequency of breakfast consumption and life satisfaction by listwise deletion method or using consumption using multiple imputations by chained, respectively. Regardless of the method used to analyze the data, the results appear to be similar.

**Fig. 2:**
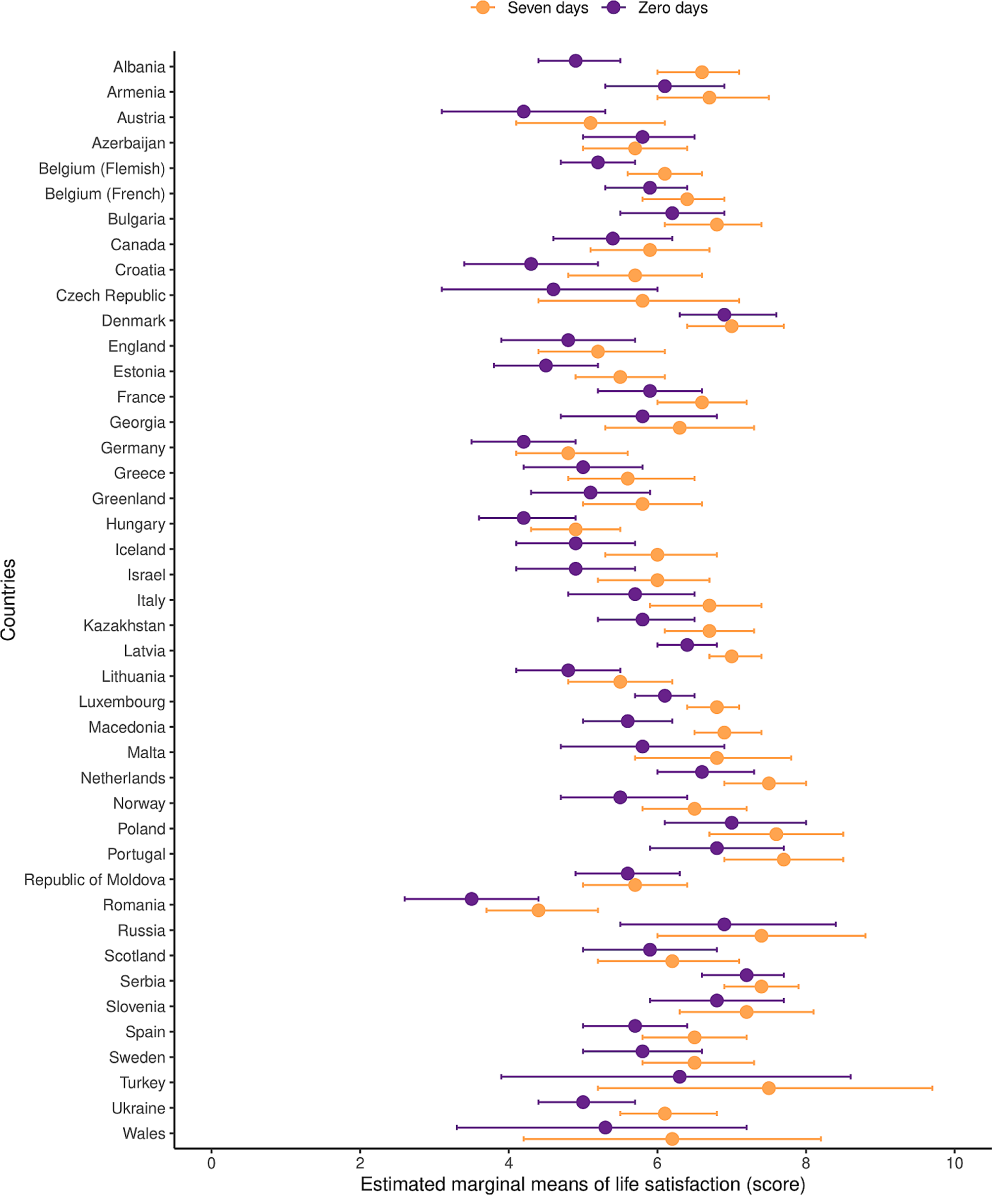
Estimated marginal means of life satisfaction according to the two extreme categories of frequency of breakfast (“zero days” versus “seven days”) for school-going children and adolescents aged 10–17 years by country. Adjusted for sex, age group, socioeconomic status (SES), fruit consumption, vegetable consumption, soft drink consumption, sweet consumption, breakfast consumption, frequency of family meals, physical activity, and body mass index. Information of the participants from one to six days of breakfast is not shown in this figure

Figure 2 displays the sensitivity analysis showing the estimated marginal mean of participants’ life satisfaction score for the extreme categories of the frequency of breakfast (i.e., “zero days” versus “seven days”) for each country. Despite inconsistencies across countries, in all 42 countries examined, the estimated marginal mean of life satisfaction score was lower in participants who no breakfast than for those who had breakfast daily. In addition, the highest estimated marginal mean of life satisfaction score was identified in those participants from Portugal who had breakfast every day (M = 7.7; 95% CI 6.9 to 8.5 points). Conversely, the lowest estimated marginal mean of life satisfaction was observed in those participants from Romania who no breakfast (M = 3.5; 95% CI 2.6 to 4.4 points). Estimated marginal means of life satisfaction based on the full frequency of breakfast consumption for each country can be found in Table S3.

## Discussion

Overall, these findings indicated that a higher frequency of breakfast consumption is associated with greater reported life satisfaction in an almost linear relationship, based on large and representative samples of school-going children and adolescents examined. The highest life satisfaction score was identified in participants who had breakfast daily, whereas the lowest life satisfaction score was observed in those participants who never had breakfast. These results are in line with a previous study which identified several behavioral factors associated with low life satisfaction in boys and girls, such as having breakfast less than daily [33]. Similarly, it has been identified observed that Iranian children and adolescents who had breakfast, lunch, and dinner 5–7 times per week reported significantly higher life satisfaction than did others [26]. There are several possible explanations for this finding.

First, one potential explanation for the observed findings could be the established association between the frequency of breakfast consumption and mood. The scientific literature has consistently highlighted the relationship between skipping breakfast and negative moods in adolescents [15, 16, 19, 43–45]. A meta-analysis found that skipping breakfast was associated with higher odds of having stress, depression, and psychological distress across all age groups, with a specific mention of anxiety in adolescents [16]. This notion is supported by another study which reported that Spanish adolescents with a higher percentage of breakfast skippers exhibited significantly lower health-related quality of life [15]. Furthermore, it has been observed that students who consumed breakfast daily, along with adopting other healthy habits, such as a high daily intake of fruits and vegetables and having at least three meals, reported the highest levels of happiness [43]. Another study reported an inverse relationship between the frequency of breakfast consumption and somatic symptoms (i.e., tiredness, sleep-problems, breathing, nausea, appetite, headache, fever) among Korean children, where increased breakfast consumption was associated with decreased somatic symptoms [45]. These somatic symptoms can significantly impact an individual’s daily life, and they are often associated with somatic symptom disorder when they cause excessive and disproportionate worry and anxiety (among others) [46]. Lastly, evidence suggests that eating breakfast, especially at home, is associated with lower odds of psychosocial behavioral problems in children and adolescents [22]. These collective findings underscore the potential impact of breakfast habits on mood and psychological well-being in this population.

A second potential explanation for the observed results could be the role of essential nutrients, vitamins, and minerals from breakfast. This meal presents an opportunity for adolescents to obtain crucial nutrients required for their overall health and well-being [47, 48]. During adolescence, the intake of essential nutrients through a nutritious breakfast is particularly important, supporting growth and development during this critical stage of life [16, 49]. A systematic review with meta-analysis highlights the association between breakfast consumption and improved macronutrient intake, healthier food and beverage consumption, and overall well-being in children and adolescents [48]. A balanced diet rich in fruits, vegetables, whole grains, lean proteins, and healthy fats nurtures the well-being of adolescents [16, 50]. Given that breakfast can serve as a source of these essential nutrients, life satisfaction could be influenced by the nutritional content of breakfast. However, it is important to note that this hypothesis should be interpreted with caution, as the study did not specifically examine the quality of breakfast, and these essential nutrients can also be obtained from other meals, such as lunch and dinner.

On the other hand, a third possible explanation for these findings could be associated with the establishment of healthy habits and routines. Consistent consumption of breakfast may be linked to an overall healthier lifestyle [19, 51]. Adolescents who regularly eat breakfast might be more inclined to adopt other healthy behaviors such as maintaining a nutritious diet, engaging in regular physical activity, limiting screen time, and ensuring optimal sleep duration, all of which contribute to their overall well-being [14, 18, 19, 51]. Consequently, a healthy lifestyle may lead to greater life satisfaction. Moreover, incorporating breakfast into a morning routine can provide structure to an adolescent’s day, potentially aiding effective time management [52]. Having a set time for breakfast encourages punctuality and organization, setting a positive tone for the remainder of the day [53]. Research emphasizes the benefits of consistent and predictable family environments for healthy development, with family routines playing a crucial role in adolescents’ long-term development [54]. The establishment of routines and structures could positively impact organization and time management, thereby contributing to greater life satisfaction [55]. In this context, the routine of having breakfast could be a key element in fostering positive habits and organization, ultimately influencing children’s and adolescents’ life satisfaction.

Additionally, a fourth potential explanation could be related to the relationship between breakfast consumption and academic performance. Several studies have shown that regular breakfast intake improves academic performance in adolescents [20, 53, 56]. Consuming an adequate breakfast provides the necessary energy and nutrients for optimal cognitive functioning and enhances concentration, memory, and learning ability [56, 57]. Studies have also shown that breakfast consumption, compared to fasting, has a short-term positive effect on domain-specific cognition, with tasks requiring attention, executive function, and memory being more reliably facilitated by breakfast consumption [53]. Moreover, another study observed that those who reported more frequent breakfast consumption and family meals were more likely to perceive their school performance as higher than their peers [20]. There is also a recognized connection between academic performance and life satisfaction in adolescents, with some studies suggesting a positive relationship between these two variables [58, 59]. Given these findings, it is possible to hypothesize that the habit of eating breakfast is associated with higher academic performance, which in turn contributes to increased life satisfaction in adolescents.

Our study identified inconsistencies in life satisfaction means among countries, which might be influenced by diverse cultures and lifestyles, such as various eating habits. Preferences and types of food consumed for breakfast, as well as the significance attributed to it, can differ greatly across cultures [49, 60]. These cultural variations might have contributed to the observed differences in life satisfaction outcomes. Additionally, individual differences among people within each country, including personal preferences, health conditions, and lifestyle choices, may have impacted life satisfaction scores and may not have been adequately controlled for in the study [61–63]. Furthermore, external influences like economic performance, economic crises, political instability, and public health issues can affect life satisfaction independently of breakfast habits [64–67]. For instance, higher national economic performance has been linked to greater life satisfaction [65]. However, the relationship between life satisfaction and gross domestic product differs in poor and rich countries due to increased aspirations for higher income [66]. Economic crises also seem to have a greater impact on life satisfaction in low-trust countries than in high-trust ones. Despite these possible cultural and economic factors, our results show that, in all the countries examined, reported life satisfaction is systematically higher in those who eat breakfast daily than in those who never eat breakfast, which adds robustness to these findings.

It is crucial to interpret the findings of this study while considering specific limitations. First, the cross-sectional design of the study restricted the ability to establish a causal relationship among the observed outcomes. Second, the use of self-reported information by adolescents could introduce recall bias and social desirability. However, this limitation is inherent to the use of questionnaires. Consequently, future prospective observational studies are essential to explore whether the frequency of breakfast consumption is genuinely associated with adolescent life satisfaction. Third, the use of concise questions aimed at reducing participant burden resulted in a lack of in-depth data on the variables under examination. The employment of a more detailed measure could potentially yield additional insights for each item and offer details on other aspects related to breakfast, such as its quality [22, 48]. Fourth, the assessment of well-being using a single subjective item (e.g., life satisfaction) may limit the understanding of how individuals experience various aspects of their lives that are crucial to critical outcomes [68]. Future studies on well-being should incorporate all major components, including both hedonic and eudaimonic aspects, rather than simplify it to a single item measuring life satisfaction, income, or happiness [68–70]. On a positive note, the study benefits from a large and representative sample of adolescents from 42 countries, thereby enhancing the external validity of the findings.

## Conclusions

There is a nearly linear relationship between higher frequency of breakfast and greater life satisfaction in children and adolescents. Considering the potential health advantages associated with breakfast during this critical age phase, these findings underscore the importance of promoting breakfast consumption among children and adolescents. However, the causal direction of the association is not entirely clear and that other factors that have not been considered could influence life satisfaction, this recommendation should be interpreted with caution.

## Electronic supplementary material

Below is the link to the electronic supplementary material.

Supplementary Material 1

## Data Availability

No datasets were generated or analysed during the current study.

## References

[1] Diener E, Lucas RE, Oishi S. Sujective well-being: the science of happiness and life satisfaction. In: Handbook of positive psychology. New York, NY, US: Oxford University Press; 2002. p. 463–73.

[2] Tian L, Wang D, Huebner ES. Development and validation of the brief adolescents’ subjective well-being in school scale (BASWBSS). Soc Indic Res. 2015;120(2):615–34.

[3] Thompson EJ, Stafford J, Moltrecht B, Huggins CF, Kwong ASF, Shaw RJ, et al. Psychological distress, depression, anxiety, and life satisfaction following COVID-19 infection: evidence from 11 UK longitudinal population studies. Lancet Psychiatry. 2022;9(11):894–906. 36244359 10.1016/S2215-0366(22)00307-8 PMC9560745

[4] González-Carrasco M, Casas F, Malo S, Viñas F, Dinisman T. Changes with age in Subjective Well-being through the adolescent years: differences by gender. J Happiness Stud. 2017;18(1):63–88.

[5] Piko BF. Adolescent life satisfaction: Association with Psychological, School-Related, Religious and socially supportive factors. Children. 2023;10(7):1176. 37508673 10.3390/children10071176 PMC10378027

[6] Azpiazu Izaguirre L, Fernández AR, Palacios EG. Adolescent life satisfaction explained by social support, emotion regulation, and Resilience. Front Psychol. 2021;12:694183. 10.3389/fpsyg.2021.694183 PMC8496342 34630207

[7] Orben A, Lucas RE, Fuhrmann D, Kievit RA. Trajectories of adolescent life satisfaction. R Soc Open Sci. 2022;9(8):211808. 35937913 10.1098/rsos.211808 PMC9346371

[8] Daly M. Cross-national and longitudinal evidence for a rapid decline in life satisfaction in adolescence. J Adolesc. 2022;94(3):422–34. 35390206 10.1002/jad.12037

[9] Morrish L, Chin TC, Rikard N, Sigley-Taylor P, Vella-Brodrick D. The role of physiological and subjective measures of emotion regulation in predicting adolescent wellbeing. Int J Wellbeing. 2019;9(2):66–89.

[10] Diener E, Heintzelman SJ, Kushlev K, Tay L, Wirtz D, Lutes LD, et al. Findings all psychologists should know from the new science on subjective well-being. Can Psychol / Psychologie Canadienne. 2017;58(2):87–104.

[11] Ross DA, Hinton R, Melles-Brewer M, Engel D, Zeck W, Fagan L, et al. Adolescent Well-Being: a definition and conceptual Framework. J Adolesc Health. 2020;67(4):472–6. 32800426 10.1016/j.jadohealth.2020.06.042 PMC7423586

[12] Otto C, Reiss F, Voss C, Wüstner A, Meyrose AK, Hölling H, et al. Mental health and well-being from childhood to adulthood: design, methods and results of the 11-year follow-up of the BELLA study. Eur Child Adolesc Psychiatry. 2021;30(10):1559–77. 32918625 10.1007/s00787-020-01630-4 PMC8505294

[13] Organisation for Economic Cooperation and Development. Health at a glance 2021: OECD indicators [Internet]. OECD. 2021 [cited 2024 Jan 15]. (Health at a Glance). Available from: https://www.oecd-ilibrary.org/social-issues-migration-health/health-at-a-glance-2021_ae3016b9-en.

[14] López-Gil JF, Tremblay MS, Tapia-Serrano MÁ, Tárraga-López PJ, Brazo-Sayavera J. Meeting 24 h Movement Guidelines and Health-Related Quality of Life in Youths during the COVID-19 Lockdown. Appl Sci. 2022;12(16):8056.

[15] Jiménez-López E, Mesas AE, Bizzozero-Peroni B, Fernández-Rodríguez R, Garrido-Miguel M, Victoria-Montesinos D, et al. Clustering of Mediterranean dietary patterns linked with health-related quality of life in adolescents: the EHDLA study. Eur J Pediatr. 2023;182(9):4113–21. 10.1007/s00431-023-05069-y 37410113

[16] Zahedi H, Djalalinia S, Sadeghi O, Zare Garizi F, Asayesh H, Payab M, et al. Breakfast consumption and mental health: a systematic review and meta-analysis of observational studies. Nutr Neurosci. 2022;25(6):1250–64. 33314992 10.1080/1028415X.2020.1853411

[17] O’Neil A, Quirk SE, Housden S, Brennan SL, Williams LJ, Pasco JA, et al. Relationship between diet and mental health in children and adolescents: a systematic review. Am J Public Health. 2014;104(10):e31–42. 25208008 10.2105/AJPH.2014.302110 PMC4167107

[18] López-Gil JF, Roman‐Viñas B, Aznar S, Tremblay MS. Meeting 24‐h movement guidelines: prevalence, correlates, and associations with socioemotional behavior in Spanish minors. Scand Med Sci Sports. 2022;32(5):881–91. 10.1111/sms.14132 PMC9303223 35090196

[19] López-Gil JF, García-Hermoso A. Adherence to the Mediterranean diet and subjective well-being among Chilean children. Appetite. 2022;172:105974. 35181381 10.1016/j.appet.2022.105974

[20] López-Gil JF, Mesas AE, Álvarez-Bueno C, Pascual-Morena C, Saz-Lara A, Cavero-Redondo I. Association between eating habits and perceived school performance: a cross-sectional study among 46,455 adolescents from 42 countries. Front Nutr. 2022;9:797415. 10.3389/fnut.2022.797415 PMC8852839 35187033

[21] Rani R, Dharaiya CN, Singh B. Importance of not skipping breakfast: a review. Int J Food Sci Technol. 2021;56(1):28–38.

[22] López-Gil JF, Smith L, López-Bueno R, Tárraga-López PJ. Breakfast and psychosocial behavioural problems in young population: the role of status, place, and habits. Front Nutr. 2022;9:871238. 10.3389/fnut.2022.871238 PMC9445130 36082031

[23] Giovannini M, Verduci E, Scaglioni S, Salvatici E, Bonza M, Riva E, et al. Breakfast: a good habit, not a repetitive custom. J Int Med Res. 2008;36(4):613–24. 18652755 10.1177/147323000803600401

[24] Peña-Jorquera H, Campos-Núñez V, Sadarangani KP, Ferrari G, Jorquera-Aguilera C, Cristi-Montero C. Breakfast: a crucial meal for adolescents’ cognitive performance according to their nutritional status. The cogni-action project. Nutrients. 2021;13(4):1320. 10.3390/nu13041320 PMC8073030 33923639

[25] Lundqvist M, Vogel NE, Levin LÅ. Effects of eating breakfast on children and adolescents: a systematic review of potentially relevant outcomes in economic evaluations. Food & Nutrition Research [Internet]. 2019 [cited 2024 Jan 15];63(0). Available from: http://www.foodandnutritionresearch.net/index.php/fnr/article/view/1618. 10.29219/fnr.v63.1618 PMC6744840 31548838

[26] Kelishadi R, Qorbani M, Heshmat R, Motlagh ME, Magoul A, Mansourian M, et al. Determinants of life satisfaction in Iranian children and adolescents: the CASPIAN-IV study. Child Adolesc Ment Health. 2018;23(3):228–34. 32677304 10.1111/camh.12239

[27] Moor I, Lampert T, Rathmann K, Kuntz B, Kolip P, Spallek J, et al. Explaining educational inequalities in adolescent life satisfaction: do health behaviour and gender matter? Int J Public Health. 2014;59(2):309–17. 24368542 10.1007/s00038-013-0531-9

[28] O’Sullivan TA, Robinson M, Kendall GE, Miller M, Jacoby P, Silburn SR, et al. A good-quality breakfast is associated with better mental health in adolescence. Public Health Nutr. 2009;12(2):249–58. 19026092 10.1017/S1368980008003935

[29] Pearson N, Atkin AJ, Biddle SJ, Gorely T, Edwardson C. Patterns of adolescent physical activity and dietary behaviours. Int J Behav Nutr Phys Act. 2009;6(1):45. 19624822 10.1186/1479-5868-6-45 PMC2718856

[30] Scheffers FR, Boer JMA, Verschuren WMM, Verheus M, van der Schouw YT, Sluijs I, et al. Pure fruit juice and fruit consumption and the risk of CVD: the European Prospective Investigation into Cancer and Nutrition–Netherlands (EPIC-NL) study. Br J Nutr. 2019;121(3):351–9. 10.1017/S0007114518003380 PMC6390400 30428938

[31] Papoutsou S, Briassoulis G, Hadjigeorgiou C, Savva SC, Solea T, Hebestreit A, et al. The combination of daily breakfast consumption and optimal breakfast choices in childhood is an important public health message. Int J Food Sci Nutr. 2014;65(3):273–9. 24512299 10.3109/09637486.2013.854750

[32] Woods N, Seabrook JA, Haines J, Stranges S, Minaker L, O’Connor C, et al. Breakfast consumption and diet quality of teens in southwestern Ontario. Curr Dev Nutr. 2023;7(2):100003. 37180078 10.1016/j.cdnut.2022.100003 PMC10111595

[33] Sincovich A, Moller H, Smithers L, Brushe M, Lassi ZS, Brinkman SA, et al. Prevalence of breakfast skipping among children and adolescents: a cross-sectional population level study. BMC Pediatr. 2022;22(1):220. 35459164 10.1186/s12887-022-03284-4 PMC9034546

[34] Esquius L, Aguilar-Martínez A, Bosque-Prous M, González-Casals H, Bach-Faig A, Colillas-Malet E, et al. Social inequalities in breakfast consumption among adolescents in Spain: the DESKcohort project. Nutrients. 2021;13(8):2500. 34444661 10.3390/nu13082500 PMC8401108

[35] Inchey J, Currie D, Cosma A, Samdal O. Health behaviour in school-aged children (HBSC) study protocol: background, methodology and mandatory items for the 2017/18 survey. St Andrews [Internet]. 2018 [cited 2024 Jan 15]. (CAHRU). Available from: https://hbsc.org/publications/survey-protocols/.

[36] Cantril H. The pattern of human concerns [Internet]. Rutgers University Press; 1965. Available from: https://books.google.com.ec/books?id=AWlqAAAAMAAJ.

[37] Hobza V, Hamrik Z, Bucksch J, De Clercq B. The family affluence scale as an indicator for socioeconomic status: validation on regional income differences in the Czech Republic. Int J Environ Res Public Health. 2017;14(12):1540. 10.3390/ijerph14121540 PMC5750958 29292773

[38] Boyce W, Torsheim T, Currie C, Zambon A. The family affluence scale as a measure of national wealth: validation of an adolescent self-report measure. Soc Indic Res. 2006;78:473–87.

[39] Jonsson KR, Bailey CK, Corell M, Löfstedt P, Adjei NK. Associations between dietary behaviours and the mental and physical well-being of Swedish adolescents. Child Adolesc Psychiatry Ment Health. 2024;18(1):43. 38555430 10.1186/s13034-024-00733-z PMC10981827

[40] Meyer S, Weidmann R, Grob A. The mirror’s curse: weight perceptions mediate the link between physical activity and life satisfaction among 727,865 teens in 44 countries. J Sport Health Sci. 2021;10(1):48–54. 10.1016/j.jshs.2020.01.002 PMC7856557 33518016

[41] van Buuren S, Groothuis-Oudshoorn K. Mice: multivariate imputation by chained equations in R. J Stat Soft [Internet]. 2011 [cited 2024 Jan 15];45(3). Available from: http://www.jstatsoft.org/v45/i03/.

[42] White IR, Royston P, Wood AM. Multiple imputation using chained equations: issues and guidance for practice. Statist Med. 2011;30(4):377–99. 21225900 10.1002/sim.4067

[43] Lesani A, Mohammadpoorasl A, Javadi M, Esfeh JM, Fakhari A. Eating breakfast, fruit and vegetable intake and their relation with happiness in college students. Eat Weight Disord. 2016;21(4):645–51. 26928281 10.1007/s40519-016-0261-0

[44] Zalazar-Jaime MF, Moretti LS, Medrano LA. Contribution of academic satisfaction judgments to subjective well-being. Front Psychol. 2022;13:772346. 35668989 10.3389/fpsyg.2022.772346 PMC9163815

[45] Lim SI, Jeong S. The relationship between the frequency of breakfast consumption, conversation with parents, and somatic symptoms in children: a three-wave latent growth model. Int J Environ Res Public Health. 2022;19(19):12975. 10.3390/ijerph191912975 PMC9564638 36232274

[46] Sardesai A, Muneshwar KN, Bhardwaj M, Goel DB. The importance of early diagnosis of somatic symptom disorder: a case report. Cureus [Internet]. 2023 [cited 2024 May 22]. Available from: https://www.cureus.com/articles/174384-the-importance-of-early-diagnosis-of-somatic-symptom-disorder-a-case-report. 10.7759/cureus.44554 PMC10544785 37790046

[47] Preziosi P, Galan P, Deheeger M, Yacoub N, Drewnowski A, Hercberg S. Breakfast type, daily nutrient intakes and vitamin and mineral status of French children, adolescents and adults. J Am Coll Nutr. 1999;18(2):171–8. 10.1080/07315724.1999.10718846 10204834

[48] Giménez-Legarre N, Miguel-Berges ML, Flores-Barrantes P, Santaliestra-Pasías AM, Moreno LA. Breakfast characteristics and its association with daily micronutrients intake in children and adolescents–a systematic review and meta-analysis. Nutrients. 2020;12(10):3201. 10.3390/nu12103201 PMC7589686 33092061

[49] Gibney M, Barr S, Bellisle F, Drewnowski A, Fagt S, Livingstone B, et al. Breakfast in human nutrition: the international breakfast research initiative. Nutrients. 2018;10(5):559. 10.3390/nu10050559 PMC5986439 29723985

[50] Soliman AT, Alaaraj N, Noor Hamed NH, Alyafei F, Ahmed S, Shaat M, et al. Review nutritional interventions during adolescence and their possible effects. Acta Biomedica Atenei Parmensis. 2022;93(1):e2022087. 10.23750/abm.v93i1.12789 PMC8972883 35315384

[51] Corder K, Winpenny E, Love R, Brown HE, White M, Sluijs E. Change in physical activity from adolescence to early adulthood: a systematic review and meta-analysis of longitudinal cohort studies. Br J Sports Med. 2019;53(8):496–503. 28739834 10.1136/bjsports-2016-097330 PMC6250429

[52] Arlinghaus KR, Johnston CA. The importance of creating habits and routine. Am J Lifestyle Med. 2019;13(2):142–4. 30800018 10.1177/1559827618818044 PMC6378489

[53] Adolphus K, Lawton CL, Champ CL, Dye L. The effects of breakfast and breakfast composition on cognition in children and adolescents: a systematic review. Adv Nutr. 2016;7(3):590S–612S. 10.3945/an.115.010256 PMC4863264 27184287

[54] Barton AW, Brody GH, Yu T, Kogan SM, Chen E, Ehrlich KB. The profundity of the everyday: family routines in adolescence predict development in young adulthood. J Adolesc Health. 2019;64(3):340–6. 30392861 10.1016/j.jadohealth.2018.08.029 PMC9389627

[55] Heintzelman SJ, King LA. Routines and meaning in life. Pers Soc Psychol Bull. 2019;45(5):688–99. 30226411 10.1177/0146167218795133

[56] Peña-Jorquera H, Martínez-Flores R, Espinoza-Puelles JP, López-Gil JF, Ferrari G, Zapata-Lamana R, et al. Adolescents with a favorable Mediterranean-style-based pattern show higher cognitive and academic achievement: a cluster analysis—the cogni-action project. Nutrients. 2024;16(5):608. 10.3390/nu16050608 PMC10934130 38474736

[57] Puri S, Shaheen M, Grover B. Nutrition and cognitive health: a life course approach. Front Public Health. 2023;11:1023907. 37050953 10.3389/fpubh.2023.1023907 PMC10083484

[58] Crede J, Wirthwein L, McElvany N, Steinmayr R. Adolescents’ academic achievement and life satisfaction: the role of parents’ education. Front Psychol. 2015;6(52):1–8. 25691877 10.3389/fpsyg.2015.00052 PMC4315030

[59] Kim B, Jeong J. Dynamics of adolescents’ life satisfaction and effect of class rank percentile: evidence from Korean panel data. J Econ Psychol. 2017;59:8–28.

[60] Lazzeri G, Ciardullo S, Spinelli A, Pierannunzio D, Dzielska A, Kelly C, et al. The correlation between adolescent daily breakfast consumption and socio-demographic: trends in 23 European countries participating in the health behaviour in school-aged children study (2002–2018). Nutrients. 2023;15(11):2453. 10.3390/nu15112453 PMC10255377 37299415

[61] Phulkerd S, Thapsuwan S, Chamratrithirong A, Gray RS. Influence of healthy lifestyle behaviors on life satisfaction in the aging population of Thailand: a national population-based survey. BMC Public Health. 2021;21(1):43. 33407252 10.1186/s12889-020-10032-9 PMC7789197

[62] Choi YK, Joshanloo M, Lee JH, Lee HS, Lee HP, Song J. Understanding key predictors of life satisfaction in a nationally representative sample of Koreans. IJERPH. 2023;20(18):6745. 10.3390/ijerph20186745 PMC10530390 37754605

[63] Gschwandtner A, Jewell S, Kambhampati US. Lifestyle and life satisfaction: the role of delayed gratification. J Happiness Stud. 2022;23(3):1043–72.

[64] Clench-Aas J, Bergande I, Nes RB, Holte A. Trust buffers against reduced life satisfaction when faced with financial crisis. Front Psychol. 2021;12:632585. 10.3389/fpsyg.2021.632585 PMC8264375 34248740

[65] Deaton A. Income, health, and well-being around the world: evidence from the Gallup World Poll. J Econ Perspect. 2008;22(2):53–72. 10.1257/jep.22.2.53 PMC2680297 19436768

[66] Proto E, Rustichini A. A reassessment of the relationship between GDP and life satisfaction. PLoS One. 2013;8(11):e79358. 10.1371/journal.pone.0079358 PMC3842267 24312179

[67] Böhnke P. Does society matter? Life satisfaction in the enlarged Europe. Soc Indic Res. 2008;87(2):189–210.

[68] Ruggeri K, Garcia-Garzon E, Maguire Á, Matz S, Huppert FA. Well-being is more than happiness and life satisfaction: a multidimensional analysis of 21 countries. Health Qual Life Outcomes. 2020;18(1):192. 32560725 10.1186/s12955-020-01423-y PMC7304199

[69] Allin P, Hand DJ. New statistics for Old?—measuring the wellbeing of the UK. J R Stat Soc Ser A Stat Soc. 2017;180(1):3–43.

[70] Galbraith ED, Barrington-Leigh C, Miñarro S, Álvarez-Fernández S, Attoh EMNAN, Benyei P, et al. High life satisfaction reported among small-scale societies with low incomes. Proc Natl Acad Sci USA. 2024;121(7):e2311703121. 10.1073/pnas.2311703121 PMC10873637 38315863

