# Supplementary material for: Is the frequency of breakfast consumption associated with life satisfaction in children and adolescents? A cross-sectional study with 154,151 participants from 42 countries
Source: Nutr J. 2024 Jul 16;23:78. doi: 10.1186/s12937-024-00979-5 (PMC11251324; doi:10.1186/s12937-024-00979-5)
Supplement: Supplementary file 1 — Supplementary Material 1 [file 12937_2024_979_MOESM1_ESM.docx]

**Is the frequency of breakfast consumption associated with life satisfaction in children and adolescents? A cross-sectional study with 154,151 participants from 42 countries**

*López-Gil et al.*

Supplementary material

##

## **Table S1.** Full result of the generalized linear model assessing the association between frequency of breakfast consumption and life satisfaction of the study participants by listwise deletion method (*N* = 154,151).

| **Predictors** |  | **Outcome** |  |
| --- | --- | --- | --- |
|  |  | **Life satisfaction (score)** |  |
|  |  | **Univariable**  ***B* (95% CI, *p*-value)** | **Multilevel**  ***B* (95% CI, *p*-value)** |
| **Specific individual average effects** |  |  |  |
| Frequency of breakfast consumption | 0 days | Reference | Reference |
|  | 1 day | 0.16 (0.10 to 0.23, *p*<0.001) | 0.17 (0.11 to 0.23, *p*<0.001) |
|  | 2 days | 0.59 (0.54 to 0.65, *p*<0.001) | 0.44 (0.39 to 0.49, *p*<0.001) |
|  | 3 days | 0.63 (0.57 to 0.70, *p*<0.001) | 0.41 (0.35 to 0.47, *p*<0.001) |
|  | 4 days | 0.63 (0.57 to 0.69, *p*<0.001) | 0.40 (0.34 to 0.46, *p*<0.001) |
|  | 5 days | 0.73 (0.67 to 0.79, *p*<0.001) | 0.50 (0.44 to 0.55, *p*<0.001) |
|  | 6 days | 0.81 (0.75 to 0.87, *p*<0.001) | 0.52 (0.46 to 0.57, *p*<0.001) |
|  | 7 days | 1.19 (1.14 to 1.24, *p*<0.001) | 0.75 (0.70 to 0.80, *p*<0.001) |
| Age | Per one year | -0.21 (-0.22 to -0.21, *p*<0.001) | -0.14 (-0.14 to 0.13, *p*<0.001) |
| Sex | Boys | Reference | Reference |
|  | Girls | -0.27 (-0.29 to -0.25, *p*<0.001) | -0.21 (-0.23 to 0.19, *p*<0.001) |
| SES | Low SES | Reference | Reference |
|  | Medium SES | 0.41 (0.38 to 0.43, *p*<0.001) | 0.30 (0.27 to 0.32, *p*<0.001) |
|  | High SES | 0.75 (0.72 to 0.78, *p*<0.001) | 0.52 (0.49 to 0.55, *p*<0.001) |
| Fruits consumption | Never | Reference | Reference |
|  | Less once a week | -0.14 (-0.21 to -0.08, *p*<0.001) | -0.04 (-0.11 to 0.02, *p*=0.093) |
|  | Once a week | 0.05 (-0.01 to 0.12, *p*=0.108) | 0.07 (0.00 to 0.13, *p*=0.019) |
|  | 2-4 days a week | 0.25 (0.19 to 0.31, *p*<0.001) | 0.15 (0.09 to 0.21, *p*<0.001) |
|  | 5-6 days a week | 0.49 (0.43 to 0.55, *p*<0.001) | 0.25 (0.19 to 0.31, *p*<0.001) |
|  | Once daily | 0.62 (0.56 to 0.68, *p*<0.001) | 0.31 (0.25 to 0.37, *p*<0.001) |
|  | More than once daily | 0.84 (0.78 to 0.90, *p*<0.001) | 0.37 (0.31 to 0.43, *p*<0.001) |
| Vegetables consumption | Never | Reference | Reference |
|  | Less once a week | -0.10 (-0.16 to -0.04, *p*=0.002) | -0.04 (-0.10 to 0.01, *p*=0.073) |
|  | Once a week | 0.07 (0.01 to 0.12, *p*=0.018) | 0.04 (-0.02 to 0.09, *p*=0.094) |
|  | 2-4 days a week | 0.16 (0.11 to 0.21, *p*<0.001) | 0.06 (0.01 to 0.11, *p*=0.011) |
|  | 5-6 days a week | 0.32 (0.27 to 0.38, *p*<0.001) | 0.10 (0.05 to 0.15, *p*<0.001) |
|  | Once daily | 0.46 (0.41 to 0.51, *p*<0.001) | 0.12 (0.07 to 0.17, *p*<0.001) |
|  | More than once daily | 0.67 (0.62 to 0.72, *p*<0.001) | 0.17 (0.12 to 0.22, *p*<0.001) |
| Soft drinks consumption | Never | Reference | Reference |
|  | Less once a week | 0.02 (-0.01 to 0.06, *p*=0.120) | 0.02 (-0.01 to 0.05, *p*=0.089) |
|  | Once a week | 0.05 (0.02 to 0.08, *p*=0.003) | 0.05 (0.02 to 0.09, *p*<0.001) |
|  | 2-4 days a week | -0.08 (-0.11 to -0.05, *p*<0.001) | 0.01 (-0.02 to 0.04, *p*=0.339) |
|  | 5-6 days a week | -0.16 (-0.20 to -0.11, *p*<0.001) | -0.04 (-0.08 to 0.00, *p*=0.018) |
|  | Once daily | -0.01 (-0.05 to 0.04, *p*=0.739) | 0.06 (0.02 to 0.11, *p*=0.002) |
|  | More than once daily | -0.04 (-0.08 to -0.00, *p*=0.046) | 0.03 (-0.01 to 0.07, *p*=0.103) |
| Sweets consumption | Never | Reference | Reference |
|  | Less once a week | 0.05 (-0.01 to 0.10, *p*=0.098) | 0.07 (0.02 to 0.12, *p*=0.003) |
|  | Once a week | 0.07 (0.02 to 0.12, *p*=0.011) | 0.08 (0.03 to 0.13, *p*=0.001) |
|  | 2-4 days a week | -0.05 (-0.10 to 0.00, *p*=0.059) | 0.02 (-0.03 to 0.07, *p*=0.209) |
|  | 5-6 days a week | -0.10 (-0.15 to -0.04, *p*<0.001) | -0.01 (-0.07 to 0.04, *p*=0.287) |
|  | Once daily | 0.04 (-0.01 to 0.10, *p*=0.127) | 0.03 (-0.03 to 0.08, *p*=0.164) |
|  | More than once daily | 0.05 (-0.01 to 0.10, *p*=0.093) | -0.02 (-0.08 to 0.03, *p*=0.185) |
| Frequency of family meals | Never | Reference | Reference |
|  | Less often | 0.67 (0.60 to 0.74, *p*<0.001) | 0.45 (0.38 to 0.52, *p*<0.001) |
|  | About once a week | 0.99 (0.92 to 1.06, *p*<0.001) | 0.73 (0.66 to 0.79, *p*<0.001) |
|  | Most days | 1.35 (1.28 to 1.42, *p*<0.001) | 0.96 (0.89 to 1.02, *p*<0.001) |
|  | Every day | 1.76 (1.70 to 1.82, *p*<0.001) | 1.26 (1.20 to 1.32, *p*<0.001) |
| Weekly physical activity ^†^ | Per one day | 0.15 (0.14 to 0.15, *p*<0.001) | 0.07 (0.07 to 0.08, *p*<0.001) |
| Body mass index | Per one kg/m^2^ | -0.06 (-0.07 to -0.06, *p*<0.001) | -0.02 (-0.02 to 0.02, *p*<0.001) |
| **General contextual effects** |  |  |  |
| Country variance |  |  | 0.12 (0.08 to 0.20) |
| ICC (%) |  |  | 3.77% (2.84% to 4.76%) |
| **Model performance** |  |  |  |
| Marginal *R^2^* |  |  | 0.119 |
| Conditional *R*^2^ |  |  | 0.153 |

*B*, unstandardized beta coefficient; CI, confidence interval; ICC, intraclass correlation coefficient; *R*^2^, coefficient of determination; SD, standard deviation; SES, socioeconomic status. ^†^ At least 60 minutes a day.

**Figure S1.** Estimated marginal means of life satisfaction based on the frequency of breakfast consumption using multiple imputations by chained methods. Data expressed as dots (means) and lines (95% confidence interval). Adjusted for sex, age group, socioeconomic status, fruit consumption, vegetable consumption, soft drink consumption, sweet consumption, frequency of family meals, physical activity, and body mass index.

## ****

## **Table S2.** Full result of the generalized linear model assessing the association between frequency of breakfast consumption and life satisfaction of the study participants by listwise deletion method using multiple imputations by chained methods (*N* = 240,951).

| **Predictors** |  | **Outcome** |  |
| --- | --- | --- | --- |
|  |  | **Life satisfaction (score)** |  |
|  |  | **Univariable**  ***B* (95% CI, *p*-value)** | **Multilevel**  ***B* (95% CI, *p*-value)** |
| **Specific individual average effects** |  |  |  |
| Frequency of breakfast consumption | 0 days | Reference | Reference |
|  | 1 day | 0.21 (0.16 to 0.26, *p*<0.001) | 0.18 (0.14 to 0.23, *p*<0.001) |
|  | 2 days | 0.65 (0.60 to 0.69, *p*<0.001) | 0.45 (0.41 to 0.49, *p*<0.001) |
|  | 3 days | 0.72 (0.67 to 0.77, *p*<0.001) | 0.46 (0.41 to 0.51, *p*<0.001) |
|  | 4 days | 0.71 (0.67 to 0.76, *p*<0.001) | 0.45 (0.40 to 0.50, *p*<0.001) |
|  | 5 days | 0.80 (0.75 to 0.84, *p*<0.001) | 0.52 (0.47 to 0.56, *p*<0.001) |
|  | 6 days | 0.91 (0.86 to 0.95, *p*<0.001) | 0.57 (0.53 to 0.61, *p*<0.001) |
|  | 7 days | 1.28 (1.24 to 1.32, p*<*0.001) | 0.80 (0.76 to 0.84, *p*<0.001) |
| Age | Per one year | -0.21 (-0.22 to -0.21, *p*<0.001) | -0.14 (-0.14 to 0.13, *p*<0.001) |
| Sex | Boys | Reference | Reference |
|  | Girls | -0.26 (-0.27 to -0.24, *p*<0.001) | -0.20 (-0.21 to 0.18, *p*<0.001) |
| SES | Low SES | Reference | Reference |
|  | Medium SES | 0.42 (0.40 to 0.44, *p*<0.001) | 0.28 (0.26 to 0.30, *p*<0.001) |
|  | High SES | 0.75 (0.72 to 0.77, *p*<0.001) | 0.49 (0.47 to 0.52, *p*<0.001) |
| Fruits consumption | Never | Reference | Reference |
|  | Less once a week | -0.07 (-0.13 to -0.02, *p*=0.005) | -0.05 (-0.10 to 0.00, *p*=0.021) |
|  | Once a week | 0.15 (0.10 to 0.20, *p*<0.001) | 0.09 (0.04 to 0.13, *p*<0.001) |
|  | 2-4 days a week | 0.33 (0.29 to 0.38, *p*<0.001) | 0.15 (0.11 to 0.20, *p*<0.001) |
|  | 5-6 days a week | 0.56 (0.51 to 0.61, *p*<0.001) | 0.25 (0.20 to 0.29, *p*<0.001) |
|  | Once daily | 0.69 (0.64 to 0.74, *p*<0.001) | 0.31 (0.26 to 0.36, *p*<0.001) |
|  | More than once daily | 0.90 (0.85 to 0.94, *p*<0.001) | 0.38 (0.33 to 0.43, *p*<0.001) |
| Vegetables consumption | Never | Reference | Reference |
|  | Less once a week | -0.06 (-0.11 to -0.01, *p*=0.012) | -0.04 (-0.09 to 0.00, *p*=0.030) |
|  | Once a week | 0.11 (0.07 to 0.15, *p*<0.001) | 0.03 (-0.01 to 0.07, *p*=0.086) |
|  | 2-4 days a week | 0.19 (0.16 to 0.23, *p*<0.001) | 0.04 (0.01 to 0.08, *p*=0.011) |
|  | 5-6 days a week | 0.36 (0.32 to 0.39, *p*<0.001) | 0.08 (0.04 to 0.12, *p*<0.001) |
|  | Once daily | 0.48 (0.44 to 0.52, *p*<0.001) | 0.08 (0.05 to 0.12, *p*<0.001) |
|  | More than once daily | 0.68 (0.64 to 0.72, *p*<0.001) | 0.13 (0.09 to 0.17, *p*<0.001) |
| Soft drinks consumption | Never | Reference | Reference |
|  | Less once a week | 0.02 (-0.00 to 0.05, *p*=0.081) | 0.01 (-0.01 to 0.03, *p*=0.201) |
|  | Once a week | 0.05 (0.02 to 0.08, *p*<0.001) | 0.04 (0.02 to 0.07, *p*=0.001) |
|  | 2-4 days a week | -0.07 (-0.09 to -0.04, *p*<0.001) | 0.01 (-0.02 to 0.03, *p*=0.264) |
|  | 5-6 days a week | -0.15 (-0.19 to -0.12, *p*<0.001) | -0.04 (-0.07 to 0.01, *p*=0.006) |
|  | Once daily | -0.05 (-0.09 to -0.01, *p*=0.007) | 0.03 (-0.00 to 0.07, *p*=0.032) |
|  | More than once daily | -0.09 (-0.12 to -0.05, *p*<0.001) | 0.02 (-0.02 to 0.05, *p*=0.164) |
| Sweets consumption | Never | Reference | Reference |
|  | Less once a week | 0.06 (0.02 to 0.11, *p*=0.004) | 0.06 (0.02 to 0.10, *p*=0.002) |
|  | Once a week | 0.10 (0.06 to 0.14, *p*<0.001) | 0.08 (0.04 to 0.12, *p*<0.001) |
|  | 2-4 days a week | 0.00 (-0.04 to 0.04, *p*=0.967) | 0.03 (-0.01 to 0.07, *p*=0.067) |
|  | 5-6 days a week | -0.03 (-0.08 to 0.01, *p*=0.119) | 0.00 (-0.04 to 0.04, *p*=0.479) |
|  | Once daily | 0.09 (0.05 to 0.14, *p*<0.001) | 0.04 (-0.00 to 0.08, *p*=0.037) |
|  | More than once daily | 0.05 (0.01 to 0.10, *p*=0.020) | -0.03 (-0.07 to 0.02, *p*=0.111) |
| Frequency of family meals | Never | Reference | Reference |
|  | Less often | 0.70 (0.64 to 0.75, *p*<0.001) | 0.46 (0.41 to 0.51, *p*<0.001) |
|  | About once a week | 1.03 (0.97 to 1.08, *p*<0.001) | 0.73 (0.68 to 0.78, *p*<0.001) |
|  | Most days | 1.36 (1.31 to 1.41, *p*<0.001) | 0.94 (0.90 to 0.99, *p*<0.001) |
|  | Every day | 1.77 (1.73 to 1.82, *p*<0.001) | 1.25 (1.20 to 1.30, *p*<0.001) |
| Weekly physical activity ^†^ | Per one day | 0.15 (0.14 to 0.15, *p*<0.001) | 0.08 (0.07 to 0.08, *p*<0.001) |
| Body mass index | Per one kg/m^2^ | -0.07 (-0.07 to -0.06, *p*<0.001) | -0.02 (-0.02 to 0.02, *p*<0.001) |
| **General contextual effects** |  |  |  |
| Country variance |  |  | 0.11 (0.08 to 0.17) |
| ICC (%) |  |  | 3.26% (2.8% to 3.8%) |
| **Model performance** |  |  |  |
| Marginal *R^2^* |  |  | 0.117 |
| Conditional *R*^2^ |  |  | 0.146 |

*B*, unstandardized beta coefficient; CI, confidence interval; ICC, intraclass correlation coefficient; *R*^2^, coefficient of determination; SD, standard deviation; SES, socioeconomic status. ^†^ At least 60 minutes a day.

**Table S3**. Estimated marginal means of life satisfaction based on the frequency of breakfast consumption by listwise deletion method for each country.

| **Country** | **Frequency of breakfast** | **Participants (%)** | **M** | **95% LLCI** | **95% ULCI** |  |
| --- | --- | --- | --- | --- | --- | --- |
| Albania | 0 days | 69 (1.3) | 4.9 | 4.4 | 5.5 |  |
|  | 1 day | 57 (0.8) | 5.5 | 4.9 | 6.1 |  |
|  | 2 days | 246 (1.4) | 5.9 | 5.3 | 6.5 |  |
|  | 3 days | 52 (0.7) | 5.8 | 5.2 | 6.4 |  |
|  | 4 days | 99 (1.1) | 5.8 | 5.2 | 6.4 |  |
|  | 5 days | 113 (0.9) | 6.0 | 5.4 | 6.6 |  |
|  | 6 days | 73 (0.5) | 6.3 | 5.7 | 6.9 |  |
|  | 7 days | 561 (0.7) | 6.6 | 6.0 | 7.1 |  |
| Armenia | 0 days | 101 (1.8) | 6.1 | 5.3 | 6.9 |  |
|  | 1 day | 129 (1.8) | 6.5 | 5.7 | 7.3 |  |
|  | 2 days | 460 (2.6) | 6.6 | 5.8 | 7.4 |  |
|  | 3 days | 138 (1.8) | 6.4 | 5.6 | 7.2 |  |
|  | 4 days | 192 (2.2) | 6.5 | 5.7 | 7.3 |  |
|  | 5 days | 228 (1.8) | 6.6 | 5.8 | 7.4 |  |
|  | 6 days | 224 (1.6) | 6.5 | 5.7 | 7.3 |  |
|  | 7 days | 1495 (1.8) | 6.7 | 6.0 | 7.5 |  |
| Austria | 0 days | 250 (4.5) | 4.2 | 3.1 | 5.3 |  |
|  | 1 day | 301 (4.2) | 4.6 | 3.6 | 5.7 |  |
|  | 2 days | 716 (4.0) | 4.7 | 3.7 | 5.7 |  |
|  | 3 days | 177 (2.3) | 4.8 | 3.8 | 5.9 |  |
|  | 4 days | 181 (2.1) | 4.6 | 3.5 | 5.7 |  |
|  | 5 days | 297 (2.3) | 4.8 | 3.7 | 5.8 |  |
|  | 6 days | 248 (1.8) | 4.8 | 3.7 | 5.8 |  |
|  | 7 days | 1320 (1.6) | 5.1 | 4.1 | 6.1 |  |
| Azerbaijan | 0 days | 318 (5.8) | 5.8 | 5.0 | 6.5 |  |
|  | 1 day | 187 (2.6) | 5.2 | 4.5 | 6.0 |  |
|  | 2 days | 493 (2.8) | 5.4 | 4.7 | 6.1 |  |
|  | 3 days | 230 (3.0) | 5.6 | 5.0 | 6.3 |  |
|  | 4 days | 166 (1.9) | 5.5 | 4.8 | 6.2 |  |
|  | 5 days | 187 (1.5) | 5.6 | 4.9 | 6.3 |  |
|  | 6 days | 210 (1.5) | 5.3 | 4.7 | 6.0 |  |
|  | 7 days | 2135 (2.6) | 5.7 | 5.0 | 6.4 |  |
| Belgium (Flemish) | 0 days | 70 (1.3) | 5.2 | 4.7 | 5.7 |  |
|  | 1 day | 100 (1.4) | 5.5 | 5.0 | 6.0 |  |
|  | 2 days | 205 (1.2) | 5.8 | 5.2 | 6.3 |  |
|  | 3 days | 106 (1.4) | 5.9 | 5.3 | 6.4 |  |
|  | 4 days | 165 (1.9) | 5.8 | 5.2 | 6.4 |  |
|  | 5 days | 249 (1.9) | 6.0 | 5.5 | 6.6 |  |
|  | 6 days | 365 (2.6) | 6.0 | 5.5 | 6.6 |  |
|  | 7 days | 2254 (2.8) | 6.1 | 5.6 | 6.6 |  |
| Belgium (French) | 0 days | 106 (1.9) | 5.9 | 5.3 | 6.4 |  |
|  | 1 day | 131 (1.8) | 6.0 | 5.4 | 6.5 |  |
|  | 2 days | 338 (1.9) | 6.2 | 5.7 | 6.8 |  |
|  | 3 days | 127 (1.7) | 6.2 | 5.6 | 6.8 |  |
|  | 4 days | 154 (1.7) | 6.1 | 5.6 | 6.7 |  |
|  | 5 days | 212 (1.7) | 6.3 | 5.8 | 6.9 |  |
|  | 6 days | 237 (1.7) | 6.3 | 5.7 | 6.8 |  |
|  | 7 days | 1721 (2.1) | 6.4 | 5.8 | 6.9 |  |
| Bulgaria | 0 days | 94 (1.7) | 6.2 | 5.5 | 6.9 |  |
|  | 1 day | 146 (2.1) | 6.1 | 5.4 | 6.8 |  |
|  | 2 days | 404 (2.3) | 6.5 | 5.9 | 7.2 |  |
|  | 3 days | 294 (3.8) | 6.3 | 5.6 | 7.0 |  |
|  | 4 days | 360 (4.1) | 6.5 | 5.8 | 7.1 |  |
|  | 5 days | 507 (4.0) | 6.4 | 5.8 | 7.1 |  |
|  | 6 days | 508 (3.6) | 6.7 | 6.1 | 7.4 |  |
|  | 7 days | 1657 (2.0) | 6.8 | 6.1 | 7.4 |  |
| Canada | 0 days | 257 (4.7) | 5.4 | 4.6 | 6.2 |  |
|  | 1 day | 380 (5.3) | 5.5 | 4.7 | 6.3 |  |
|  | 2 days | 612 (3.4) | 5.7 | 5.0 | 6.5 |  |
|  | 3 days | 342 (4.4) | 5.6 | 4.8 | 6.4 |  |
|  | 4 days | 492 (5.6) | 5.7 | 4.9 | 6.5 |  |
|  | 5 days | 684 (5.3) | 5.8 | 5.0 | 6.6 |  |
|  | 6 days | 812 (5.8) | 5.7 | 4.9 | 6.5 |  |
|  | 7 days | 3633 (4.5) | 5.9 | 5.1 | 6.7 |  |
| Croatia | 0 days | 142 (2.6) | 4.3 | 3.4 | 5.2 |  |
|  | 1 day | 201 (2.8) | 5.0 | 4.1 | 5.9 |  |
|  | 2 days | 536 (3.0) | 5.2 | 4.3 | 6.1 |  |
|  | 3 days | 229 (3.0) | 5.1 | 4.2 | 6.0 |  |
|  | 4 days | 371 (4.2) | 5.2 | 4.3 | 6.2 |  |
|  | 5 days | 458 (3.6) | 5.3 | 4.4 | 6.2 |  |
|  | 6 days | 477 (3.4) | 5.4 | 4.5 | 6.3 |  |
|  | 7 days | 1935 (2.4) | 5.7 | 4.8 | 6.6 |  |
| Denmark | 0 days | 44 (0.8) | 6.9 | 6.3 | 7.6 |  |
|  | 1 day | 57 (0.8) | 6.8 | 6.2 | 7.5 |  |
|  | 2 days | 146 (0.8) | 7.0 | 6.4 | 7.7 |  |
|  | 3 days | 107 (1.4) | 6.9 | 6.2 | 7.5 |  |
|  | 4 days | 88 (1.0) | 6.6 | 5.9 | 7.3 |  |
|  | 5 days | 187 (1.5) | 7.0 | 6.3 | 7.7 |  |
|  | 6 days | 210 (1.5) | 6.3 | 5.7 | 7.0 |  |
|  | 7 days | 1652 (2.0) | 7.0 | 6.4 | 7.7 |  |
| England | 0 days | 18 (0.3) | 4.8 | 3.9 | 5.7 |  |
|  | 1 day | 22 (0.3) | 4.7 | 3.8 | 5.6 |  |
|  | 2 days | 23 (0.1) | 4.9 | 4.0 | 5.8 |  |
|  | 3 days | 17 (0.2) | 5.2 | 4.3 | 6.1 |  |
|  | 4 days | 13 (0.1) | 5.0 | 4.1 | 5.9 |  |
|  | 5 days | 24 (0.2) | 5.0 | 4.1 | 5.9 |  |
|  | 6 days | 39 (0.3) | 5.0 | 4.1 | 5.9 |  |
|  | 7 days | 140 (0.2) | 5.2 | 4.4 | 6.1 |  |
| Estonia | 0 days | 120 (2.2) | 4.5 | 3.8 | 5.2 |  |
|  | 1 day | 151 (2.1) | 4.6 | 4.0 | 5.3 |  |
|  | 2 days | 387 (2.2) | 5.1 | 4.5 | 5.7 |  |
|  | 3 days | 189 (2.5) | 4.8 | 4.1 | 5.5 |  |
|  | 4 days | 204 (2.3) | 5.0 | 4.3 | 5.7 |  |
|  | 5 days | 338 (2.6) | 5.0 | 4.4 | 5.7 |  |
|  | 6 days | 389 (2.8) | 5.1 | 4.5 | 5.7 |  |
|  | 7 days | 2110 (2.6) | 5.5 | 4.9 | 6.1 |  |
| France | 0 days | 313 (5.7) | 5.9 | 5.2 | 6.6 |  |
|  | 1 day | 294 (4.1) | 5.9 | 5.2 | 6.5 |  |
|  | 2 days | 610 (3.4) | 6.0 | 5.4 | 6.6 |  |
|  | 3 days | 266 (3.5) | 6.1 | 5.4 | 6.7 |  |
|  | 4 days | 348 (3.9) | 6.1 | 5.5 | 6.8 |  |
|  | 5 days | 558 (4.4) | 6.3 | 5.6 | 6.9 |  |
|  | 6 days | 569 (4.0) | 6.4 | 5.7 | 7.0 |  |
|  | 7 days | 4007 (5.0) | 6.6 | 6.0 | 7.2 |  |
| Georgia | 0 days | 148 (2.7) | 5.8 | 4.7 | 6.8 |  |
|  | 1 day | 96 (1.3) | 5.8 | 4.7 | 6.8 |  |
|  | 2 days | 309 (1.7) | 6.0 | 5.0 | 7.1 |  |
|  | 3 days | 150 (2.0) | 6.0 | 4.9 | 7.1 |  |
|  | 4 days | 235 (2.7) | 5.7 | 4.6 | 6.8 |  |
|  | 5 days | 219 (1.7) | 6.2 | 5.1 | 7.2 |  |
|  | 6 days | 174 (1.2) | 6.2 | 5.2 | 7.2 |  |
|  | 7 days | 1134 (1.4) | 6.3 | 5.3 | 7.3 |  |
| Germany | 0 days | 116 (2.1) | 4.2 | 3.5 | 4.9 |  |
|  | 1 day | 236 (3.3) | 4.2 | 3.4 | 4.9 |  |
|  | 2 days | 618 (3.5) | 4.4 | 3.7 | 5.1 |  |
|  | 3 days | 135 (1.8) | 4.5 | 3.7 | 5.2 |  |
|  | 4 days | 131 (1.5) | 4.5 | 3.7 | 5.2 |  |
|  | 5 days | 247 (1.9) | 4.5 | 3.7 | 5.2 |  |
|  | 6 days | 280 (2.0) | 4.6 | 3.9 | 5.4 |  |
|  | 7 days | 1821 (2.3) | 4.8 | 4.1 | 5.6 |  |
| Greece | 0 days | 103 (1.9) | 5.0 | 4.2 | 5.8 |  |
|  | 1 day | 207 (2.9) | 5.1 | 4.2 | 5.9 |  |
|  | 2 days | 678 (3.8) | 5.4 | 4.5 | 6.2 |  |
|  | 3 days | 152 (2.0) | 5.4 | 4.5 | 6.3 |  |
|  | 4 days | 222 (2.5) | 5.4 | 4.6 | 6.3 |  |
|  | 5 days | 292 (2.3) | 5.4 | 4.5 | 6.3 |  |
|  | 6 days | 281 (2.0) | 5.5 | 4.6 | 6.4 |  |
|  | 7 days | 1363 (1.7) | 5.6 | 4.8 | 6.5 |  |
| Greenland | 0 days | 25 (0.5) | 5.1 | 4.3 | 5.9 |  |
|  | 1 day | 22 (0.3) | 5.2 | 4.4 | 6.0 |  |
|  | 2 days | 37 (0.2) | 5.7 | 4.9 | 6.4 |  |
|  | 3 days | 19 (0.2) | 5.5 | 4.7 | 6.3 |  |
|  | 4 days | 11 (0.1) | 5.5 | 4.7 | 6.3 |  |
|  | 5 days | 37 (0.3) | 5.8 | 5.1 | 6.6 |  |
|  | 6 days | 34 (0.2) | 5.6 | 4.8 | 6.4 |  |
|  | 7 days | 206 (0.3) | 5.8 | 5.0 | 6.6 |  |
| Hungary | 0 days | 131 (2.4) | 4.2 | 3.6 | 4.9 |  |
|  | 1 day | 227 (3.2) | 4.4 | 3.7 | 5.0 |  |
|  | 2 days | 672 (3.8) | 4.6 | 4.0 | 5.2 |  |
|  | 3 days | 157 (2.0) | 4.4 | 3.8 | 5.1 |  |
|  | 4 days | 233 (2.6) | 4.6 | 4.0 | 5.3 |  |
|  | 5 days | 295 (2.3) | 4.6 | 4.0 | 5.2 |  |
|  | 6 days | 212 (1.5) | 4.8 | 4.1 | 5.4 |  |
|  | 7 days | 1285 (1.6) | 4.9 | 4.3 | 5.5 |  |
| Iceland | 0 days | 215 (3.9) | 4.9 | 4.1 | 5.7 |  |
|  | 1 day | 179 (2.5) | 5.4 | 4.6 | 6.2 |  |
|  | 2 days | 366 (2.1) | 5.5 | 4.7 | 6.2 |  |
|  | 3 days | 191 (2.5) | 5.4 | 4.6 | 6.2 |  |
|  | 4 days | 246 (2.8) | 5.5 | 4.6 | 6.3 |  |
|  | 5 days | 447 (3.5) | 5.7 | 4.9 | 6.4 |  |
|  | 6 days | 698 (5.0) | 5.7 | 4.9 | 6.5 |  |
|  | 7 days | 2947 (3.6) | 6.0 | 5.3 | 6.8 |  |
| Israel | 0 days | 88 (1.6) | 4.9 | 4.1 | 5.7 |  |
|  | 1 day | 265 (3.7) | 5.2 | 4.4 | 6.0 |  |
|  | 2 days | 330 (1.9) | 5.6 | 4.8 | 6.3 |  |
|  | 3 days | 155 (2.0) | 5.6 | 4.8 | 6.4 |  |
|  | 4 days | 141 (1.6) | 5.5 | 4.7 | 6.3 |  |
|  | 5 days | 165 (1.3) | 5.7 | 4.9 | 6.4 |  |
|  | 6 days | 246 (1.7) | 5.7 | 5.0 | 6.5 |  |
|  | 7 days | 1024 (1.3) | 6.0 | 5.2 | 6.7 |  |
| Italy | 0 days | 151 (2.7) | 5.7 | 4.8 | 6.5 |  |
|  | 1 day | 326 (4.6) | 6.0 | 5.2 | 6.9 |  |
|  | 2 days | 395 (2.2) | 6.1 | 5.3 | 6.8 |  |
|  | 3 days | 112 (1.5) | 6.4 | 5.6 | 7.2 |  |
|  | 4 days | 155 (1.8) | 6.2 | 5.4 | 7.0 |  |
|  | 5 days | 231 (1.8) | 6.2 | 5.4 | 7.0 |  |
|  | 6 days | 242 (1.7) | 6.1 | 5.4 | 6.9 |  |
|  | 7 days | 1788 (2.2) | 6.7 | 5.9 | 7.4 |  |
| Kazakhstan | 0 days | 92 (1.7) | 5.8 | 5.2 | 6.5 |  |
|  | 1 day | 98 (1.4) | 6.2 | 5.6 | 6.8 |  |
|  | 2 days | 251 (1.4) | 6.3 | 5.7 | 7.0 |  |
|  | 3 days | 214 (2.8) | 6.5 | 5.9 | 7.1 |  |
|  | 4 days | 220 (2.5) | 6.1 | 5.5 | 6.7 |  |
|  | 5 days | 257 (2.0) | 6.3 | 5.7 | 6.9 |  |
|  | 6 days | 365 (2.6) | 6.5 | 5.9 | 7.1 |  |
|  | 7 days | 1794 (2.2) | 6.7 | 6.1 | 7.3 |  |
| Latvia | 0 days | 122 (2.2) | 6.4 | 6.0 | 6.8 |  |
|  | 1 day | 139 (2.0) | 6.4 | 6.0 | 6.8 |  |
|  | 2 days | 460 (2.6) | 6.6 | 6.2 | 7.0 |  |
|  | 3 days | 170 (2.2) | 6.7 | 6.3 | 7.1 |  |
|  | 4 days | 213 (2.4) | 6.5 | 6.1 | 7.0 |  |
|  | 5 days | 403 (3.1) | 6.7 | 6.3 | 7.1 |  |
|  | 6 days | 360 (2.6) | 6.8 | 6.4 | 7.2 |  |
|  | 7 days | 2143 (2.7) | 7.0 | 6.7 | 7.4 |  |
| Lithuania | 0 days | 127 (2.3) | 4.8 | 4.1 | 5.5 |  |
|  | 1 day | 156 (2.2) | 4.8 | 4.1 | 5.6 |  |
|  | 2 days | 532 (3.0) | 5.2 | 4.4 | 5.9 |  |
|  | 3 days | 137 (1.8) | 5.1 | 4.3 | 5.8 |  |
|  | 4 days | 167 (1.9) | 5.0 | 4.3 | 5.8 |  |
|  | 5 days | 267 (2.1) | 5.2 | 4.5 | 5.9 |  |
|  | 6 days | 270 (1.9) | 5.2 | 4.5 | 6.0 |  |
|  | 7 days | 1468 (1.8) | 5.5 | 4.8 | 6.2 |  |
| Luxembourg | 0 days | 167 (3.0) | 6.1 | 5.7 | 6.5 |  |
|  | 1 day | 163 (2.3) | 6.2 | 5.8 | 6.6 |  |
|  | 2 days | 475 (2.7) | 6.6 | 6.3 | 7.0 |  |
|  | 3 days | 151 (2.0) | 6.4 | 6.1 | 6.8 |  |
|  | 4 days | 145 (1.6) | 6.5 | 6.2 | 6.9 |  |
|  | 5 days | 220 (1.7) | 6.6 | 6.2 | 6.9 |  |
|  | 6 days | 193 (1.4) | 6.6 | 6.3 | 7.0 |  |
|  | 7 days | 1274 (1.6) | 6.8 | 6.4 | 7.1 |  |
| Malta | 0 days | 93 (1.7) | 5.8 | 4.7 | 6.9 |  |
|  | 1 day | 105 (1.5) | 6.0 | 4.9 | 7.1 |  |
|  | 2 days | 196 (1.1) | 6.1 | 5.0 | 7.2 |  |
|  | 3 days | 64 (0.8) | 6.1 | 4.9 | 7.3 |  |
|  | 4 days | 71 (0.8) | 6.2 | 5.1 | 7.3 |  |
|  | 5 days | 127 (1.0) | 6.5 | 5.4 | 7.6 |  |
|  | 6 days | 128 (0.9) | 6.5 | 5.4 | 7.6 |  |
|  | 7 days | 562 (0.7) | 6.8 | 5.7 | 7.8 |  |
| North Macedonia | 0 days | 51 (0.9) | 5.6 | 5.0 | 6.2 |  |
|  | 1 day | 70 (1.0) | 6.3 | 5.8 | 6.9 |  |
|  | 2 days | 188 (1.1) | 6.4 | 5.9 | 7.0 |  |
|  | 3 days | 138 (1.8) | 6.6 | 6.1 | 7.1 |  |
|  | 4 days | 203 (2.3) | 6.6 | 6.1 | 7.1 |  |
|  | 5 days | 311 (2.4) | 6.6 | 6.1 | 7.1 |  |
|  | 6 days | 348 (2.5) | 6.7 | 6.2 | 7.2 |  |
|  | 7 days | 2383 (2.9) | 6.9 | 6.5 | 7.4 |  |
| Norway | 0 days | 34 (0.6) | 5.5 | 4.7 | 6.4 |  |
|  | 1 day | 44 (0.6) | 5.9 | 5.0 | 6.7 |  |
|  | 2 days | 127 (0.7) | 5.7 | 4.9 | 6.5 |  |
|  | 3 days | 71 (0.9) | 6.2 | 5.4 | 7.0 |  |
|  | 4 days | 95 (1.1) | 6.4 | 5.6 | 7 |  |
|  | 5 days | 131 (1.0) | 6.2 | 5.4 | 6.9 |  |
|  | 6 days | 206 (1.5) | 6.2 | 5.5 | 7.0 |  |
|  | 7 days | 1317 (1.6) | 6.5 | 5.8 | 7.2 |  |
| Poland | 0 days | 91 (1.6) | 7.0 | 6.1 | 8.0 |  |
|  | 1 day | 157 (2.2) | 7.5 | 6.6 | 8.5 |  |
|  | 2 days | 626 (3.5) | 7.1 | 6.2 | 8.0 |  |
|  | 3 days | 177 (2.3) | 7.2 | 6.3 | 8.2 |  |
|  | 4 days | 190 (2.2) | 7.1 | 6.2 | 8.0 |  |
|  | 5 days | 313 (2.4) | 7.5 | 6.5 | 8.4 |  |
|  | 6 days | 376 (2.7) | 7.3 | 6.4 | 8.2 |  |
|  | 7 days | 2576 (3.2) | 7.6 | 6.7 | 8.5 |  |
| Portugal | 0 days | 104 (1.9) | 6.8 | 5.9 | 7.7 |  |
|  | 1 day | 123 (1.7) | 6.7 | 5.8 | 7.6 |  |
|  | 2 days | 267 (1.5) | 7.2 | 6.3 | 8.0 |  |
|  | 3 days | 225 (2.9) | 7.2 | 6.3 | 8.0 |  |
|  | 4 days | 267 (3.0) | 6.9 | 6.1 | 7.8 |  |
|  | 5 days | 424 (3.3) | 7.4 | 6.5 | 8.2 |  |
|  | 6 days | 571 (4.1) | 7.3 | 6.5 | 8.2 |  |
|  | 7 days | 3429 (4.2) | 7.7 | 6.9 | 8.5 |  |
|  |  |  |  |  |  |  |
|  |  |  |  |  |  |  |
|  |  |  |  |  |  |  |
|  |  |  |  |  |  |  |
|  |  |  |  |  |  |  |
|  |  |  |  |  |  |  |
|  |  |  |  |  |  |  |
|  |  |  |  |  |  |  |
| Romania | 0 days | 188 (3.4) | 3.5 | 2.6 | 4.4 |  |
|  | 1 day | 177 (2.5) | 3.7 | 2.9 | 4.5 |  |
|  | 2 days | 406 (2.3) | 4.2 | 3.4 | 4.9 |  |
|  | 3 days | 155 (2.0) | 4.1 | 3.3 | 4.8 |  |
|  | 4 days | 200 (2.3) | 4.1 | 3.3 | 4.9 |  |
|  | 5 days | 236 (1.8) | 4.3 | 3.5 | 5.1 |  |
|  | 6 days | 237 (1.7) | 4.2 | 3.4 | 5.0 |  |
|  | 7 days | 1046 (1.3) | 4.4 | 3.7 | 5.2 |  |
|  |  |  |  |  |  |  |
|  |  |  |  |  |  |  |
|  |  |  |  |  |  |  |
|  |  |  |  |  |  |  |
|  |  |  |  |  |  |  |
|  |  |  |  |  |  |  |
|  |  |  |  |  |  |  |
|  |  |  |  |  |  |  |
| Scotland | 0 days | 52 (0.9) | 5.9 | 5.0 | 6.8 |  |
|  | 1 day | 68 (1.0) | 5.3 | 4.4 | 6.3 |  |
|  | 2 days | 93 (0.5) | 5.7 | 4.8 | 6.7 |  |
|  | 3 days | 37 (0.5) | 5.7 | 4.8 | 6.7 |  |
|  | 4 days | 48 (0.5) | 6.0 | 5.1 | 6.9 |  |
|  | 5 days | 95 (0.7) | 6.0 | 5.1 | 6.9 |  |
|  | 6 days | 117 (0.8) | 6.1 | 5.2 | 7.0 |  |
|  | 7 days | 743 (0.9) | 6.2 | 5.2 | 7.1 |  |
| Serbia | 0 days | 41 (0.7) | 7.2 | 6.6 | 7.7 |  |
|  | 1 day | 79 (1.1) | 7.2 | 6.7 | 7.8 |  |
|  | 2 days | 248 (1.4) | 7.3 | 6.8 | 7.8 |  |
|  | 3 days | 475 (6.2) | 7.2 | 6.7 | 7.7 |  |
|  | 4 days | 181 (2.1) | 7.1 | 6.6 | 7.6 |  |
|  | 5 days | 206 (1.6) | 7.2 | 6.7 | 7.7 |  |
|  | 6 days | 198 (1.4) | 7.0 | 6.5 | 7.5 |  |
|  | 7 days | 1876 (2.3) | 7.4 | 6.9 | 7.9 |  |
| Slovenia | 0 days | 228 (4.1) | 6.8 | 5.9 | 7.7 |  |
|  | 1 day | 312 (4.4) | 6.8 | 5.9 | 7.8 |  |
|  | 2 days | 960 (5.4) | 7.1 | 6.2 | 8.0 |  |
|  | 3 days | 365 (4.7) | 6.8 | 5.9 | 7.8 |  |
|  | 4 days | 358 (4.1) | 6.8 | 5.8 | 7.7 |  |
|  | 5 days | 496 (3.9) | 7.0 | 6.0 | 7.9 |  |
|  | 6 days | 473 (3.4) | 7.1 | 6.1 | 8.0 |  |
|  | 7 days | 2058 (2.5) | 7.2 | 6.3 | 8.1 |  |
| Spain | 0 days | 97 (1.8) | 5.7 | 5.0 | 6.4 |  |
|  | 1 day | 113 (1.6) | 5.7 | 5.0 | 6.4 |  |
|  | 2 days | 355 (2.0) | 6.1 | 5.4 | 6.8 |  |
|  | 3 days | 187 (2.4) | 6.3 | 5.6 | 7.1 |  |
|  | 4 days | 224 (2.5) | 6.3 | 5.6 | 7.0 |  |
|  | 5 days | 274 (2.1) | 6.3 | 5.7 | 7.0 |  |
|  | 6 days | 324 (2.3) | 6.1 | 5.4 | 6.8 |  |
|  | 7 days | 2245 (2.8) | 6.5 | 5.8 | 7.2 |  |
| Sweden | 0 days | 75 (1.4) | 5.8 | 5.0 | 6.6 |  |
|  | 1 day | 89 (1.3) | 6.1 | 5.3 | 6.9 |  |
|  | 2 days | 191 (1.1) | 6.2 | 5.4 | 7.0 |  |
|  | 3 days | 104 (1.4) | 6.1 | 5.3 | 6.9 |  |
|  | 4 days | 104 (1.2) | 6.5 | 5.7 | 7.3 |  |
|  | 5 days | 230 (1.8) | 6.1 | 5.3 | 6.9 |  |
|  | 6 days | 286 (2.0) | 6.3 | 5.5 | 7.1 |  |
|  | 7 days | 1856 (2.3) | 6.5 | 5.8 | 7.3 |  |
| The Czech Republic | 0 days | 338 (6.1) | 4.6 | 3.1 | 6.0 |  |
|  | 1 day | 522 (7.3) | 4.2 | 2.8 | 5.5 |  |
|  | 2 days | 1542 (8.7) | 5.2 | 3.8 | 6.5 |  |
|  | 3 days | 383 (5.0) | 4.9 | 3.5 | 6.3 |  |
|  | 4 days | 578 (6.6) | 5.3 | 3.9 | 6.6 |  |
|  | 5 days | 872 (6.8) | 5.4 | 4.0 | 6.7 |  |
|  | 6 days | 808 (5.7) | 5.2 | 3.8 | 6.5 |  |
|  | 7 days | 4918 (6.1) | 5.8 | 4.4 | 7.1 |  |
| The Netherlands | | 0 days | 33 (0.6) | 6.6 | 6.0 | 7.3 |
|  | | 1 day | 54 (0.8) | 6.9 | 6.3 | 7.5 |
|  | | 2 days | 127 (0.7) | 7.1 | 6.5 | 7.7 |
|  | | 3 days | 67 (0.9) | 7.2 | 6.6 | 7.8 |
|  | | 4 days | 96 (1.1) | 7.0 | 6.4 | 7.6 |
|  | | 5 days | 172 (1.3) | 7.1 | 6.5 | 7.7 |
|  | | 6 days | 316 (2.2) | 7.4 | 6.8 | 7.9 |
|  | | 7 days | 2374 (2.9) | 7.5 | 6.9 | 8.0 |
| The Republic of Moldova | 0 days | 97 (1.8) | 5.6 | 4.9 | 6.3 |  |
|  | 1 day | 138 (1.9) | 5.4 | 4.6 | 6.1 |  |
|  | 2 days | 307 (1.7) | 5.5 | 4.8 | 6.2 |  |
|  | 3 days | 278 (3.6) | 5.4 | 4.7 | 6.1 |  |
|  | 4 days | 290 (3.3) | 5.6 | 4.9 | 6.3 |  |
|  | 5 days | 393 (3.1) | 5.5 | 4.9 | 6.2 |  |
|  | 6 days | 407 (2.9) | 5.5 | 4.8 | 6.2 |  |
|  | 7 days | 2095 (2.6) | 5.7 | 5.0 | 6.4 |  |
| The Russian Federation | 0 days | 146 (2.6) | 6.9 | 5.5 | 8.4 |  |
|  | 1 day | 156 (2.2) | 7.3 | 5.8 | 8.7 |  |
|  | 2 days | 349 (2.0) | 7.3 | 5.9 | 8.7 |  |
|  | 3 days | 295 (3.8) | 7.5 | 6.0 | 8.9 |  |
|  | 4 days | 235 (2.7) | 7.4 | 5.9 | 8.8 |  |
|  | 5 days | 315 (2.5) | 7.3 | 5.9 | 8.7 |  |
|  | 6 days | 384 (2.7) | 7.5 | 6.0 | 8.9 |  |
|  | 7 days | 1772 (2.2) | 7.4 | 6.0 | 8.8 |  |
| Turkey | 0 days | 70 (1.3) | 6.3 | 3.9 | 8.6 |  |
|  | 1 day | 248 (3.5) | 8.0 | 5.7 | 10.3 |  |
|  | 2 days | 812 (4.6) | 7.2 | 4.9 | 9.5 |  |
|  | 3 days | 258 (3.4) | 8.1 | 5.7 | 10.6 |  |
|  | 4 days | 337 (3.8) | 7.0 | 4.5 | 9.4 |  |
|  | 5 days | 366 (2.9) | 7.2 | 4.9 | 9.4 |  |
|  | 6 days | 357 (2.5) | 7.7 | 5.4 | 10.0 |  |
|  | 7 days | 1965 (2.4) | 7.5 | 5.2 | 9.7 |  |
| Ukraine | 0 days | 108 (2.0) | 5.0 | 4.4 | 5.7 |  |
|  | 1 day | 95 (1.3) | 5.2 | 4.6 | 5.9 |  |
|  | 2 days | 343 (1.9) | 5.8 | 5.2 | 6.5 |  |
|  | 3 days | 167 (2.2) | 5.7 | 5.1 | 6.4 |  |
|  | 4 days | 182 (2.1) | 5.9 | 5.2 | 6.5 |  |
|  | 5 days | 382 (3.0) | 6.0 | 5.4 | 6.7 |  |
|  | 6 days | 392 (2.8) | 6.0 | 5.4 | 6.7 |  |
|  | 7 days | 2971 (3.7) | 6.1 | 5.5 | 6.8 |  |
| Wales | 0 days | 285 (5.2) | 5.3 | 3.3 | 7.2 |  |
|  | 1 day | 293 (4.1) | 5.0 | 2.9 | 7.1 |  |
|  | 2 days | 360 (2.0) | 5.6 | 3.4 | 7.7 |  |
|  | 3 days | 219 (2.9) | 5.7 | 3.4 | 8.0 |  |
|  | 4 days | 218 (2.5) | 6.0 | 3.7 | 8.3 |  |
|  | 5 days | 342 (2.7) | 5.2 | 3.2 | 7.3 |  |
|  | 6 days | 433 (3.1) | 5.2 | 3.1 | 7.3 |  |
|  | 7 days | 1804 (2.2) | 6.2 | 4.2 | 8.2 |  |

Adjusted for sex, age group, socioeconomic status, fruit consumption, vegetable consumption, soft drink consumption, sweet consumption, breakfast consumption, frequency of family meals, physical activity, and body mass index. M, mean; LLCI, lower limit confidence interval; ULCI, upper limit confidence interval.
